# Supplementary material for: Designing Functionally Substituted Pyridine-Carbohydrazides for Potent Antibacterial and Devouring Antifungal Effect on Multidrug Resistant (MDR) Strains
Source: Molecules. 2022 Dec 26;28(1):212. doi: 10.3390/molecules28010212 (PMC9822510; doi:10.3390/molecules28010212)
Supplement: Supplementary file 1 [file molecules-28-00212-s001.zip › molecules-2059613-supplementary.pdf]

SUPPORTING INFORMATION

Designing Functionally Substituted Pyridine-Carbohydrazides for Potent Antibacterial and Devouring Antifungal Effect on Multidrug Resistant (MDR) Strains

Farooq-Ahmad Khan [1,2,*], Sana Yaqoob [1,2], Shujaat Ali [1,2], Nimra Tanveer [1,2], Yan Wang [2,*], Sajda Ashraf [2], Khwaja Ali Hasan [3,*], Shaden A. M. Khalifa[4], Qiyang Shou [5], Zaheer Ul-Haq [6], Zi-Hua Jiang [7], Hesham R. El-Seedi [8,9,10,*]

[1] Third World Center for Science and Technology, International Center for Chemical and Biological Sciences, University of Karachi, Karachi-75270, Pakistan
[2] H.E.J. Research Institute of Chemistry, International Center for Chemical and Biological Sciences, University of Karachi, Karachi-75270, Pakistan
[3] Molecular and Structural Biology Research Unit, Department of Biochemistry, University of Karachi, Karachi-75270, Pakistan
[4] Department of Molecular Biosciences, The Wenner-Gren Institute, Stockholm University, S- 106 91 Stockholm, Sweden
[5] Second Clinical Medical College, Zhejiang Chinese Medical University, Hangzhou 310053, China
[6] Dr. Panjwani Center for Molecular Medicine and Drug Research, International Center for Chemical and Biological Sciences, University of Karachi, Karachi-75270, Pakistan
[7] Department of Chemistry, Lakehead University, 955 Oliver Road, Thunder Bay, Ontario, P7B 5E1, Canada
[8] International Research Center for Food Nutrition and Safety, Jiangsu University, Zhenjiang, 212013, China
[9] Pharmacognosy Group, Department of Pharmaceutical Biosciences, BMC, Uppsala University, Box 591, SE-751 24 Uppsala, Sweden
[10] International Joint Research Laboratory of Intelligent Agriculture and Agri-Products Processing (Jiangsu University), Jiangsu Education Department, Nanjing 210024, China
* Correspondence: farooq.khan@iccs.edu (F.K); yan.wang@iccs.edu (Y.W); ali.hasan@uok.edu.pk (K.A.H); hesham.el-seedi@farmbio.uu.se (H.R.E.-S.)

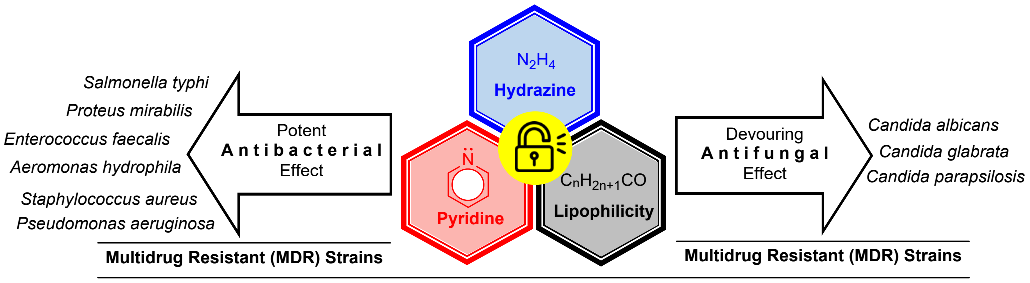

Table of contents

		Page #
1.	**Figure S1.** IR spectrum of *N*'-Acetylpridine-3-carbohydrazide (**3**)	3
2.	**Figure S2.** ^{1}H-NMR spectrum of *N*'-Acetylpridine-3-carbohydrazide (**3**)	3
3.	**Figure S3.** LR-MS (EI) spectrum of *N*'-Acetylpridine-3-carbohydrazide (**3**)	4
4.	**Figure S4.** HR-MS (EI) spectral data of *N*'-Acetylpridine-3-carbohydrazide (**3**)	4
5.	**Figure S5.** IR spectrum of *N*'-Butyrylpridine-3-carbohydrazide (**4**)	5
6.	**Figure S6.** ^{1}H NMR spectrum of N'-Butyrylpridine-3-carbohydrazide (**4**)	5
7.	**Figure S7.** ^{13}C NMR spectrum of *N*'-Butyrylpridine-3-carbohydrazide (**4**)	6
8.	**Figure S8.** LR-MS (EI) spectrum of *N*'-Butyrylpridine-3-carbohydrazide (**4**)	6
9.	**Figure S9.** HR-MS (EI) spectral data of *N*'-Butyrylpridine-3-carbohydrazide (**4**)	7
10.	**Figure S10.** IR spectrum of *N*'-Hexanoylpyridine-3-carbohydrazide (**5**)	8
11.	**Figure S11.** ^{1}H NMR spectrum of *N*'-Hexanoylpyridine-3-carbohydrazide (**5**)	8
12.	**Figure S12.** LR-MS (EI) spectrum of *N*'-Hexanoylpyridine-3-carbohydrazide (**5**)	9
13.	**Figure S13.** HR-MS (EI) spectral data of *N*'-Hexanoylpyridine-3-carbohydrazide (**5**)	10
14.	**Figure S14.** IR spectrum of *N*'-Octanoylpyridine-3-carbohydrazide (**6**)	11
15.	**Figure S15.** ^{1}H NMR spectrum of *N*'-Octanoylpyridine-3-carbohydrazide (**6**)	11
16.	**Figure S16.** LR-MS (EI) spectrum of *N*'-Octanoylpyridine-3-carbohydrazide (**6**)	12
17.	**Figure S17.** HR-MS (EI) spectral data of *N*'-Octanoylpyridine-3-carbohydrazide (**6**)	13
18.	**Figure S18.** IR spectrum of *N*'-Decanoylpyridine-3-carbohydrazide (**7**)	14
19.	**Figure S19.** ^{1}H NMR spectrum of *N*'-Decanoylpyridine-3-carbohydrazide (**7**)	14
20.	**Figure S20.** LR-MS (EI) spectrum of *N*'-Decanoylpyridine-3-carbohydrazide (**7**)	15
21.	**Figure S21.** HR-MS (EI) spectral data of *N*'-Decanoylpyridine-3-carbohydrazide (**7**)	15
22.	**Figure S22.** IR spectrum of *N*'-Acetylpyridine-4-carbohydrazide (**8**)	16
23.	**Figure S23.** ^{1}H NMR spectrum of *N*'-Acetylpyridine-4-carbohydrazide (**8**)	16
24.	**Figure S24.** LR-MS (EI) spectrum of *N*'-Acetylpyridine-4-carbohydrazide (**8**)	17
25.	**Figure S25.** HR-MS (EI) spectral data of *N*'-Acetylpyridine-4-carbohydrazide (**8**)	17
26.	**Figure S26.** IR spectrum of *N*'-Butyrylpyridine-4-carbohydrazide (**9**)	18
27.	**Figure S27.** ^{1}H NMR spectrum of *N*'-Butyrylpyridine-4-carbohydrazide (**9**)	18
28.	**Figure S28.** ^{13}C NMR spectrum of *N*'-Butyrylpyridine-4-carbohydrazide (**9**)	19
29.	**Figure S29.** LR-MS (EI) spectrum of *N*'-Butyrylpyridine-4-carbohydrazide (**9**)	19
30.	**Figure S30.** HR-MS (EI) spectral data of *N*'-Butyrylpyridine-4-carbohydrazide (**9**)	20
31.	**Figure S31.** IR spectrum of *N*'-Hexanoylpyridine-4-carbohydrazide (**10**)	20
32.	**Figure S32.** ^{1}H NMR spectrum of *N*'-Hexanoylpyridine-4-carbohydrazide (**10**)	21
33.	**Figure S33.** LR-MS (EI) spectrum of *N*'-Hexanoylpyridine-4-carbohydrazide (**10**)	21
34.	**Figure S32.** ^{1}H NMR spectrum of *N*'-Hexanoylpyridine-4-carbohydrazide (**10**)	22
35.	**Figure S33.** LR-MS (EI) spectrum of *N*'-Hexanoylpyridine-4-carbohydrazide (**10**)	23
36.	**Figure S34.** HR-MS (EI) spectral data of *N*'-Hexanoylpyridine-4-carbohydrazide (**10**)	23
37.	**Figure S35.** IR spectrum of *N*'-Octanoylpyridine-4-carbohydrazide (**11**)	24
38.	**Figure S36.** ^{1}H NMR spectrum of *N*'-Octanoylpyridine-4-carbohydrazide (**11**)	25
39.	**Figure S37.** LR-MS (EI) spectrum of *N*'-Octanoylpyridine-4-carbohydrazide (**11**)	24
40.	**Figure S38.** HR-MS (EI) spectral data of *N*'-Octanoylpyridine-4-carbohydrazide (**11**)	25
41.	**Figure S39.** IR spectrum of *N*'-Decanoylpyridine-4-carbohydrazide (**12**)	26
42	**Figure S40.** ^{1}H NMR spectrum of *N*'-Decanoylpyridine-4-carbohydrazide (**12**)	26
43.	**Figure S41.** LR-MS (EI) spectrum of *N*'-Decanoylpyridine-4-carbohydrazide (**12**)	27
44.	**Figure S42.** HR-MS (EI) spectral data of *N*'-Decanoylpyridine-4-carbohydrazide (**12**)	28
45.	Time-kill curve and hemolysis assay data of compound **4** and **6** in tabular form	31

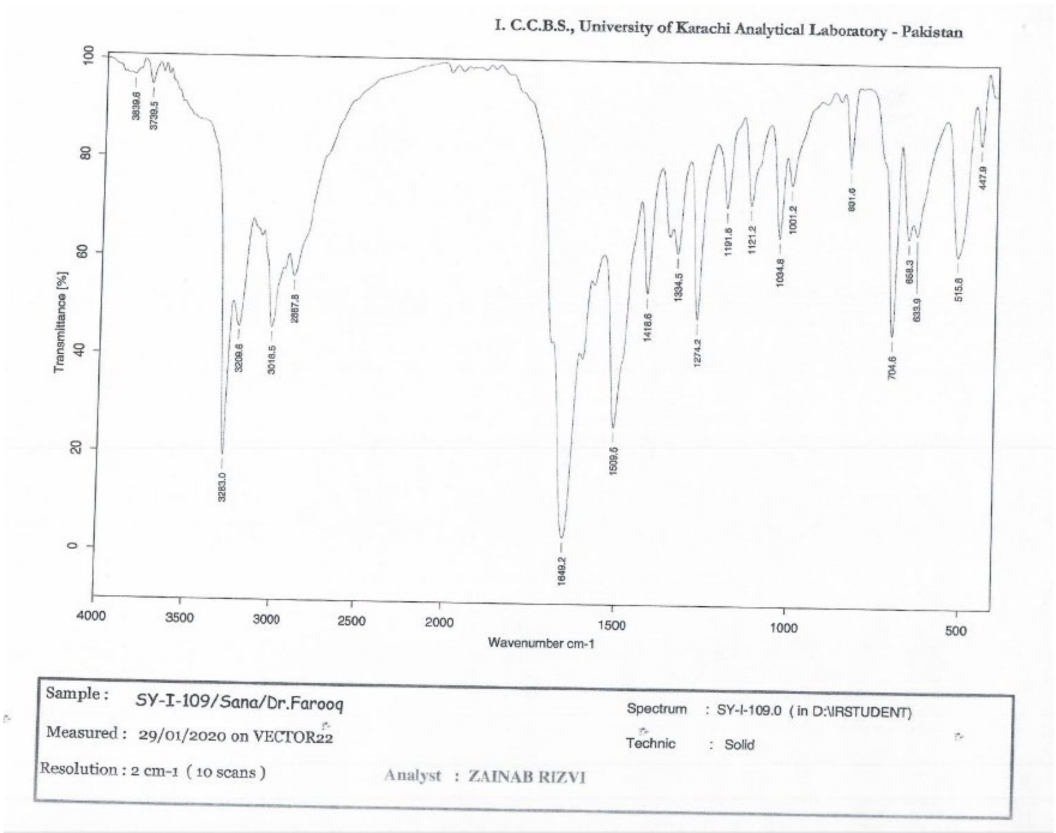

Figure S1. IR spectrum of *N*'-Acetylpridine-3-carbohydrazide (**3**)

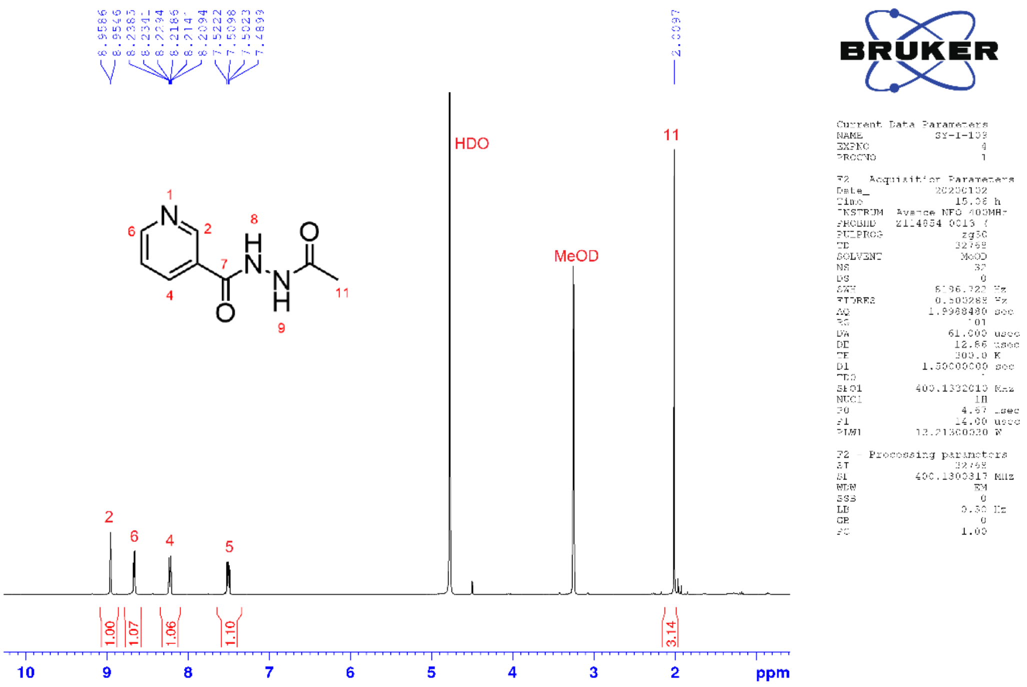

Figure S2. ^{1}H-NMR spectrum of *N*'-Acetylpridine-3-carbohydrazide (**3**)

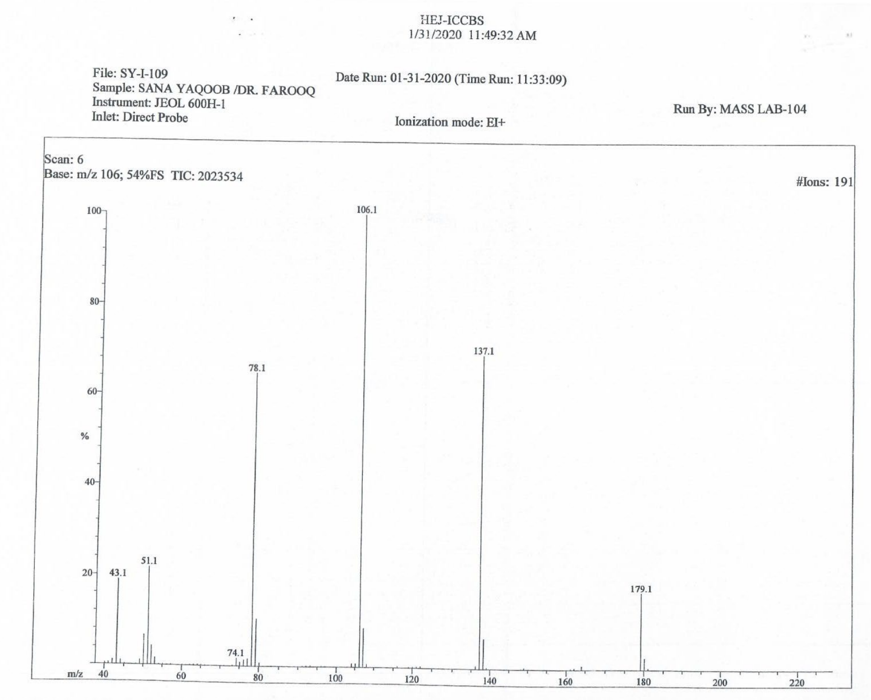

Figure S3. LR-MS (EI) spectrum of *N*'-Acetylpridine-3-carbohydrazide (**3**)

File : D:\Data\sy-i-109-190220-c9.RAW
Full ms [49.500 - 750.500] - Range: 67.000 - 750.500
Scan No. 1 of 1

Mass	Relative Intensity	Theoretical Mass	Delta [ppm]	Delta [mmu]	RDB	Composition
67.0558	1.2	67.0548	15.7	1.1	2.5	$C_5 H_7$
69.0704	3.3	69.0704	-0.5	-0.0	1.5	$C_5 H_9$
70.0772	3.8	70.0783	-14.4	-1.0	1.0	$C_5 H_{10}$
71.0853	2.0	71.0861	-10.9	-0.8	0.5	$C_5 H_{11}$
74.0468	1.4	74.0480	-16.0	-1.2	1.0	$C_2 H_6 O_1 N_2$
76.0176	15.4	76.0187	-14.2	-1.1	5.5	$C_5 H_2 N_1$
		76.0160	21.1	1.6	1.0	$C_2 H_4 O_3$
77.0255	38.5	77.0265	-14.1	-1.1	5.0	$C_5 H_3 N_1$
		77.0239	20.7	1.6	0.5	$C_2 H_5 O_3$
78.0332	68.0	78.0344	-14.8	-1.2	4.5	$C_5 H_4 N_1$
		78.0317	19.6	1.5	0.0	$C_2 H_6 O_3$
79.0402	12.4	79.0422	-25.0	-2.0	4.0	$C_5 H_5 N_1$
		79.0382	25.9	2.0	0.0	$H_5 O_2 N_3$
81.0699	1.7	81.0704	-6.6	-0.5	2.5	$C_6 H_9$
83.0851	2.1	83.0861	-11.5	-1.0	1.5	$C_6 H_{11}$
90.0344	1.4	90.0344	0.0	0.0	5.5	$C_6 H_4 N_1$
		90.0317	29.8	2.7	1.0	$C_3 H_6 O_3$
91.0415	30.5	91.0422	-7.8	-0.7	5.0	$C_6 H_5 N_1$
		91.0395	21.7	2.0	0.5	$C_3 H_7 O_3$
92.0402	4.3	92.0374	29.6	2.7	5.0	$C_5 H_4 N_2$
97.1014	1.4	97.1017	-3.3	-0.3	1.5	$C_7 H_{13}$
98.0467	2.5	98.0480	-13.5	-1.3	3.0	$C_4 H_6 O_1 N_2$
103.0306	2.4	103.0296	9.0	0.9	6.5	$C_6 H_3 N_2$
104.0382	74.1	104.0374	7.5	0.8	6.0	$C_6 H_4 N_2$
105.0570	13.1	105.0578	-7.7	-0.8	5.0	$C_7 H_7 N_1$
		105.0552	17.8	1.9	0.5	$C_4 H_9 O_3$
106.0288	67.4	106.0293	-4.9	-0.5	5.5	$C_6 H_4 O_1 N_1$
		106.0266	20.3	2.2	1.0	$C_3 H_6 O_4$
107.0342	5.8	107.0344	-2.4	-0.3	0.5	$C_3 H_7 O_4$
		107.0331	10.1	1.1	1.0	$C_1 H_5 O_3 N_3$
		107.0371	-27.5	-2.9	5.0	$C_6 H_5 O_1 N_1$
112.1258	1.1	112.1252	5.5	0.6	1.0	$C_8 H_{16}$
119.0481	6.5	119.0483	-2.2	-0.3	6.0	$C_6 H_5 N_3$
		119.0497	-13.4	-1.6	5.5	$C_8 H_7 O_1$
		119.0457	20.4	2.4	1.5	$C_3 H_7 O_3 N_2$
120.0331	2.7	120.0324	5.9	0.7	6.0	$C_6 H_4 O_1 N_2$
		120.0297	28.2	3.4	1.5	$C_3 H_6 O_4 N_1$
123.0321	1.6	123.0320	0.7	0.1	5.0	$C_6 H_5 O_2 N_1$
		123.0293	22.5	2.8	0.5	$C_3 H_7 O_5$
125.0279	1.7	125.0265	10.9	1.4	9.0	$C_9 H_3 N_1$
137.0590	24.3	137.0589	0.4	0.1	5.0	$C_6 H_7 O_1 N_3$
		137.0603	-9.4	-1.3	4.5	$C_8 H_9 O_2$
		137.0562	20.0	2.7	0.5	$C_3 H_9 O_4 N_2$
138.0622	2.1	138.0641	-13.7	-1.9	0.0	$C_3 H_{10} O_4 N_2$
146.0333	1.4	146.0328	4.0	0.6	3.0	$C_4 H_6 O_4 N_2$
		146.0354	-14.4	-2.1	7.5	$C_7 H_4 O_1 N_3$
		146.0368	-23.6	-3.4	7.0	$C_9 H_6 O_2$
147.0417	1.4	147.0406	7.4	1.1	2.5	$C_4 H_7 O_4 N_2$
		147.0433	-10.8	-1.6	7.0	$C_7 H_5 O_1 N_3$
		147.0446	-19.9	-2.9	6.5	$C_9 H_7 O_2$
149.0230	1.8	149.0225	3.0	0.4	7.0	$C_6 H_3 O_2 N_3$
		149.0239	-6.1	-0.9	6.5	$C_8 H_5 O_3$
		149.0198	20.9	3.1	2.5	$C_3 H_5 O_5 N_2$
		149.0265	-24.0	-3.6	11.0	$C_{11} H_3 N_1$
161.0573	88.0	161.0562	6.6	1.1	2.5	$C_5 H_9 O_4 N_2$
		161.0589	-10.0	-1.6	7.0	$C_8 H_7 O_1 N_3$
		161.0603	-18.3	-3.0	6.5	$C_{10} H_9 O_2$
162.0611	9.2	162.0641	-18.2	-2.9	2.0	$C_5 H_{10} O_4 N_2$
179.0688	6.6	179.0695	-3.7	-0.7	6.0	$C_8 H_9 O_2 N_3$
		179.0708	-11.2	-2.0	5.5	$C_{10} H_{11} O_3$
		179.0668	11.2	2.0	1.5	$C_5 H_{11} O_5 N_2$
		179.0735	-26.2	-4.7	10.0	$C_{13} H_9 N_1$

Figure S4. HR-MS (EI) spectral data of *N*'-Acetylpridine-3-carbohydrazide (**3**)

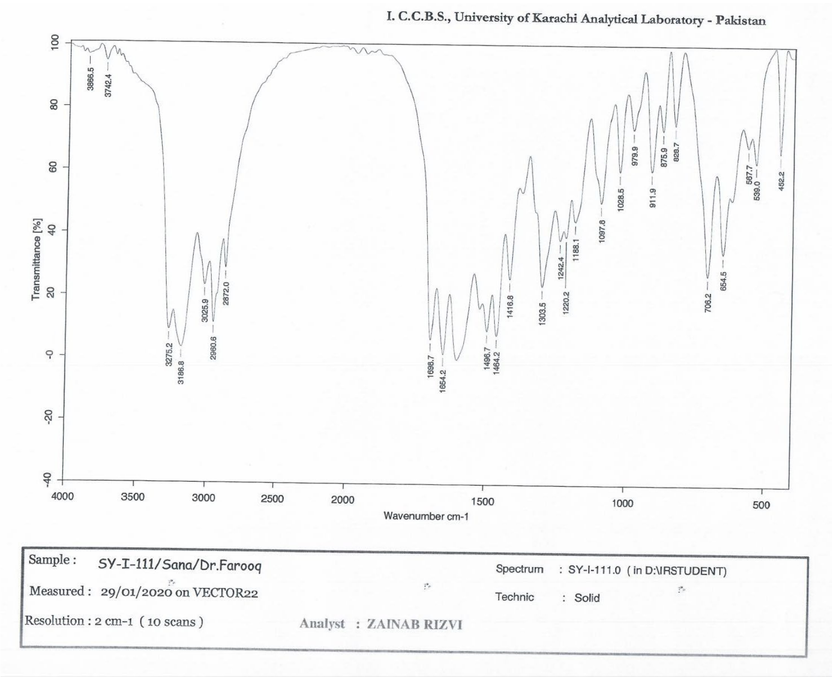

Figure S5. IR spectrum of *N'*-Butyrylpridine-3-carbohydrazide (**4**)

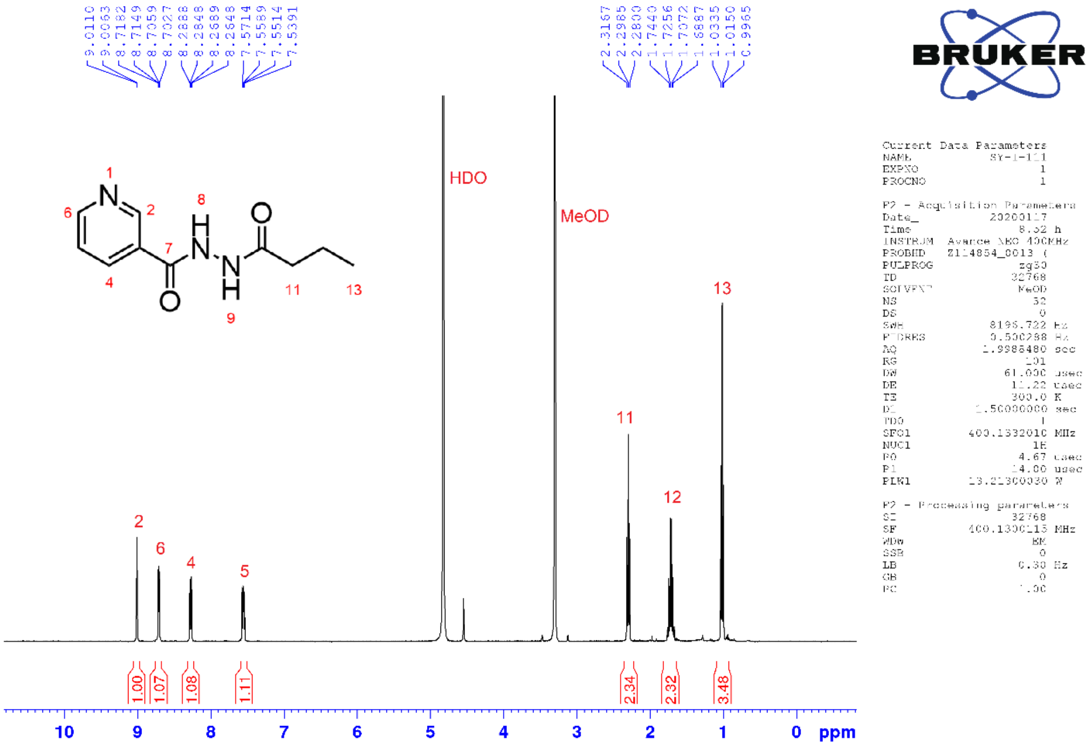

Figure S6. ¹H NMR spectrum of N'-Butyrylpridine-3-carbohydrazide (**4**)

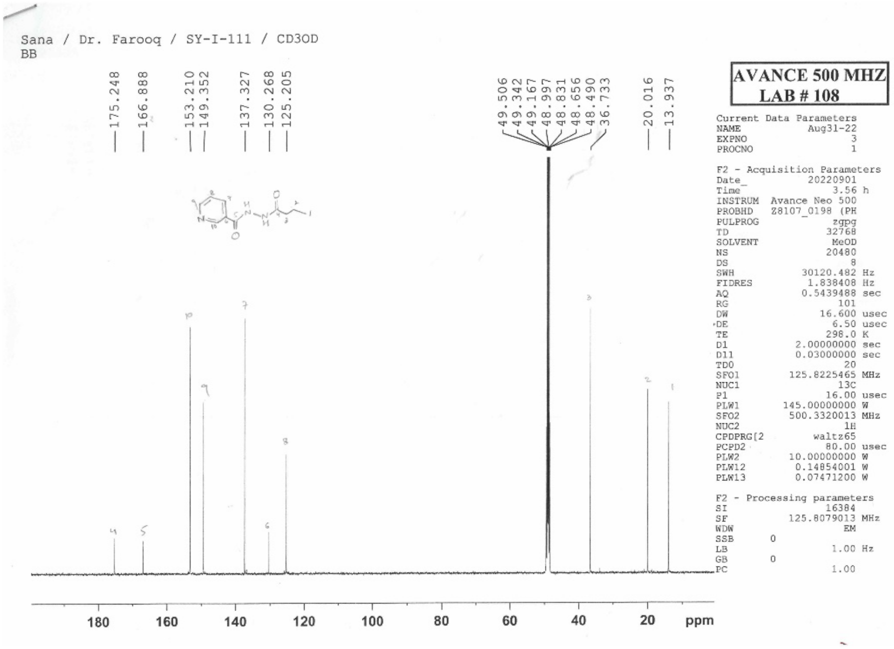

Figure S7. ^{13}C NMR spectrum of *N*'-Butyrylpridine-3-carbohydrazide (**4**)

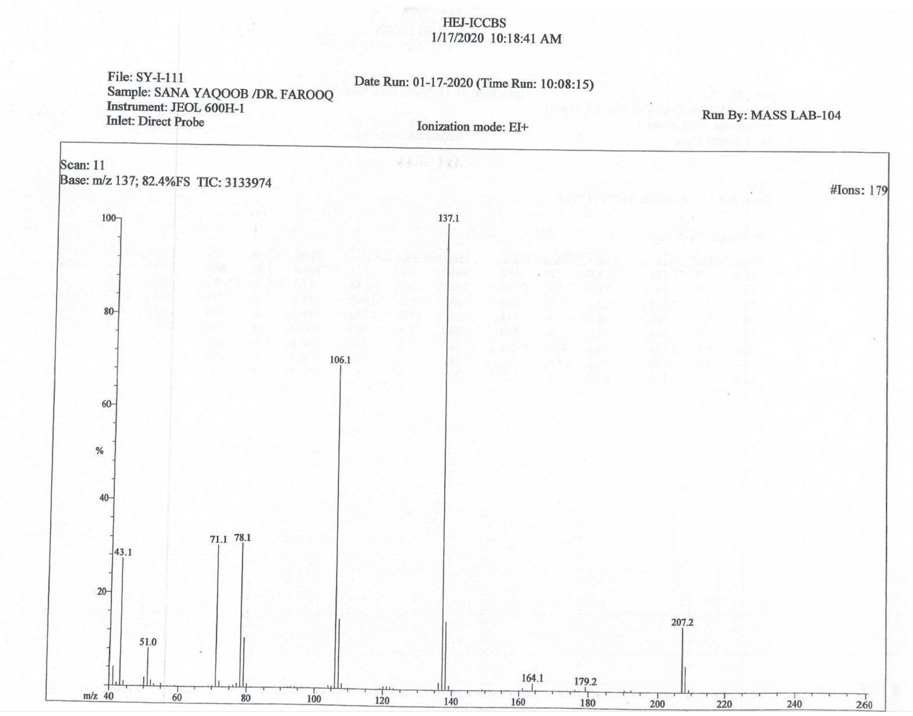

Figure S8. LR-MS (EI) spectrum of *N*'-Butyrylpridine-3-carbohydrazide (**4**)

File : D:\Data\sy-i-111_170220-c4.RAW
Full ms [49.500 - 750.500] - Range: 49.500 - 750.500
Scan No. 1 of 1

Mass	Relative Intensity	Theoretical Mass	Delta [ppm]	Delta [mmu]	RDB	Composition
50.9907	3.2					
52.0214	3.4					
52.0324	2.6	52.0313	21.1	1.1	3.0	$C_4 H_4$
53.0164	1.0					
53.0402	1.1	53.0391	19.7	1.0	2.5	$C_4 H_5$
55.0422	3.2	55.0422	0.2	0.0	2.0	$C_3 H_5 N_1$
55.0784	3.0					
57.0981	1.9					
60.0476	4.0					
62.0386	1.9	62.0368	29.9	1.9	0.0	$C_2 H_6 O_2$
63.0435	3.6	63.0433	3.5	0.2	0.0	$H_5 O_1 N_3$
64.0360	2.5					
64.0466	2.2					
65.0405	1.4	65.0391	21.4	1.4	3.5	$C_5 H_5$
69.0335	1.9	69.0340	-7.9	-0.5	2.5	$C_4 H_5 O_1$
		69.0327	11.6	0.8	3.0	$C_2 H_3 N_3$
70.0378	1.9					
70.0740	1.4					
71.0420	17.5					
72.0423	1.0					
73.0146	1.4	73.0164	-24.9	-1.8	2.0	$C_2 H_3 O_2 N_1$
74.9918	2.4					
75.9953	4.6	75.9949	4.5	0.3	6.0	$C_5 O_1$
77.0002	11.7	76.9987	19.8	1.5	1.5	$H_1 O_3 N_2$
78.0064	44.9	78.0065	-2.2	-0.2	1.0	$H_2 O_3 N_2$
79.0108	5.9					
91.0370	18.1	91.0382	-12.5	-1.1	1.0	$C_1 H_5 O_2 N_3$
		91.0395	-27.2	-2.5	0.5	$C_3 H_7 O_3$
92.0395	2.9	92.0374	22.8	2.1	5.0	$C_5 H_4 N_2$
103.0303	1.0	103.0296	6.3	0.7	6.5	$C_6 H_3 N_2$
104.0398	20.2	104.0374	23.1	2.4	6.0	$C_6 H_4 N_2$
105.0210	5.6	105.0215	-4.8	-0.5	6.0	$C_6 H_3 O_1 N_1$
		105.0188	20.7	2.2	1.5	$C_3 H_5 O_4$
105.0520	4.5	105.0538	-17.5	-1.8	1.0	$C_2 H_7 O_2 N_3$
106.0287	46.1	106.0293	-5.4	-0.6	5.5	$C_6 H_4 O_1 N_1$
		106.0266	19.9	2.1	1.0	$C_3 H_6 O_4$
107.0335	4.9	107.0331	3.7	0.4	1.0	$C_1 H_5 O_3 N_3$
		107.0344	-8.9	-0.9	0.5	$C_3 H_7 O_4$
117.0578	2.0	117.0578	-0.4	-0.0	6.0	$C_8 H_7 N_1$
		117.0552	22.5	2.6	1.5	$C_5 H_9 O_3$
118.0650	3.7	118.0657	-5.4	-0.6	5.5	$C_8 H_8 N_1$
		118.0630	17.3	2.0	1.0	$C_5 H_{10} O_3$
		118.0617	28.7	3.4	1.5	$C_3 H_8 O_2 N_3$
119.0431	9.3	119.0457	-21.6	-2.6	1.5	$C_3 H_7 O_3 N_2$
120.0378	2.0	120.0409	-26.2	-3.1	1.5	$C_2 H_6 O_3 N_3$
122.0478	1.6	122.0480	-1.6	-0.2	5.0	$C_6 H_6 O_1 N_2$
		122.0453	20.4	2.5	0.5	$C_3 H_8 O_4 N_1$
123.0315	7.8	123.0320	-4.4	-0.5	5.0	$C_6 H_5 O_2 N_1$
		123.0293	17.4	2.1	0.5	$C_3 H_7 O_5$
		123.0280	28.3	3.5	1.0	$C_1 H_5 O_4 N_3$
130.0656	1.4	130.0657	-0.9	-0.1	6.5	$C_9 H_8 N_1$
		130.0630	19.8	2.6	2.0	$C_6 H_{10} O_3$
132.0807	1.2	132.0813	-4.6	-0.6	5.5	$C_9 H_{10} N_1$
		132.0786	15.7	2.1	1.0	$C_6 H_{12} O_3$
		132.0773	25.8	3.4	1.5	$C_4 H_{10} O_2 N_3$
137.0585	17.8	137.0589	-3.2	-0.4	5.0	$C_6 H_7 O_1 N_3$
		137.0603	-13.0	-1.8	4.5	$C_8 H_9 O_2$
		137.0562	16.4	2.2	0.5	$C_3 H_9 O_4 N_2$
138.0615	2.0	138.0641	-18.5	-2.6	0.0	$C_3 H_{10} O_4 N_2$
149.0210	2.9	149.0198	7.5	1.1	2.5	$C_3 H_5 O_5 N_2$
		149.0225	-10.4	-1.6	7.0	$C_6 H_3 O_2 N_3$
		149.0239	-19.5	-2.9	6.5	$C_8 H_5 O_3$
161.0570	100.0	161.0562	5.0	0.8	2.5	$C_5 H_9 O_4 N_2$
		161.0589	-11.6	-1.9	7.0	$C_8 H_7 O_1 N_3$
		161.0603	-20.0	-3.2	6.5	$C_{10} H_9 O_2$
162.0591	8.9	162.0555	22.2	3.6	6.5	$C_9 H_8 O_2 N_1$
167.0326	1.0	167.0331	-3.1	-0.5	6.0	$C_6 H_5 O_3 N_3$
		167.0344	-11.1	-1.9	5.5	$C_8 H_7 O_4$
		167.0371	-27.2	-4.5	10.0	$C_{11} H_5 O_1 N_1$
174.0670	7.2	174.0667	1.4	0.2	7.5	$C_9 H_8 O_1 N_3$
		174.0681	-6.4	-1.1	7.0	$C_{11} H_{10} O_2$
		174.0641	16.8	2.9	3.0	$C_6 H_{10} O_4 N_2$
187.0746	3.1	187.0746	0.2	0.0	8.0	$C_{10} H_9 O_1 N_3$
		187.0759	-7.0	-1.3	7.5	$C_{12} H_{11} O_2$
		187.0719	14.5	2.7	3.5	$C_7 H_{11} O_4 N_2$
188.0808	1.9	188.0797	5.7	1.1	3.0	$C_7 H_{12} O_4 N_2$
		188.0824	-8.5	-1.6	7.5	$C_{10} H_{10} O_1 N_3$
		188.0837	-15.7	-3.0	7.0	$C_{12} H_{12} O_2$
189.0899	7.4	189.0902	-1.5	-0.3	7.0	$C_{10} H_{11} O_1 N_3$
		189.0916	-8.6	-1.6	6.5	$C_{12} H_{13} O_2$
		189.0875	12.7	2.4	2.5	$C_7 H_{13} O_4 N_2$
190.0924	1.2	190.0954	-15.5	-3.0	2.0	$C_7 H_{14} O_4 N_2$
		190.0868	29.5	5.6	6.5	$C_{11} H_{12} O_2 N_1$
		190.0980	-29.6	-5.6	6.5	$C_{10} H_{12} O_1 N_3$
207.1004	1.6	207.1008	-2.0	-0.4	6.0	$C_{10} H_{13} O_2 N_3$
		207.1021	-8.4	-1.7	5.5	$C_{12} H_{15} O_3$
		207.0981	11.0	2.3	1.5	$C_7 H_{15} O_5 N_2$
		207.1048	-21.4	-4.4	10.0	$C_{15} H_{13} N_1$

Figure S9. HR-MS (EI) spectral data of *N*'-Butyrylpridine-3-carbohydrazide (**4**)

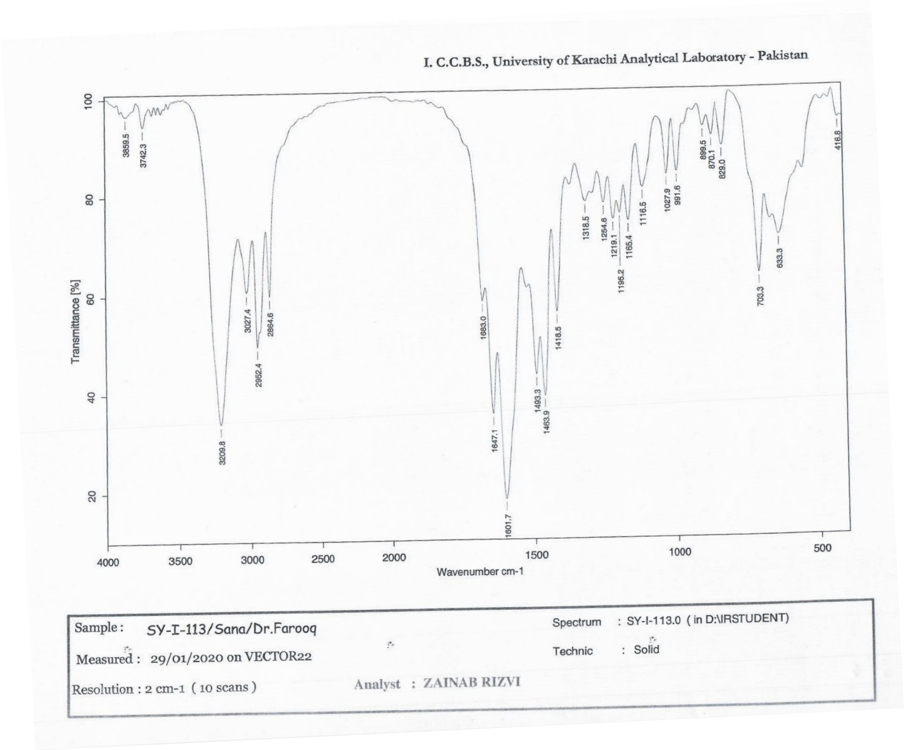

Figure S10. IR spectrum of *N'*-Hexanoylpyridine-3-carbohydrazide (**5**)

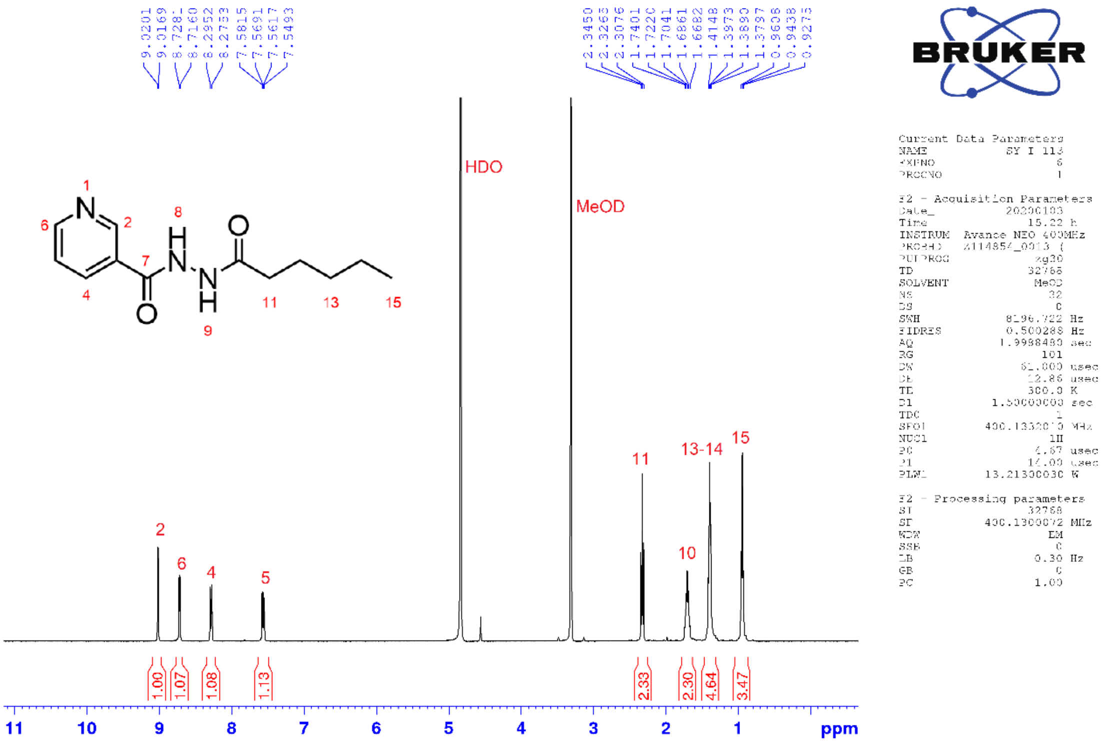

Figure S11. ^{1}H NMR spectrum of *N'*-Hexanoylpyridine-3-carbohydrazide (**5**)

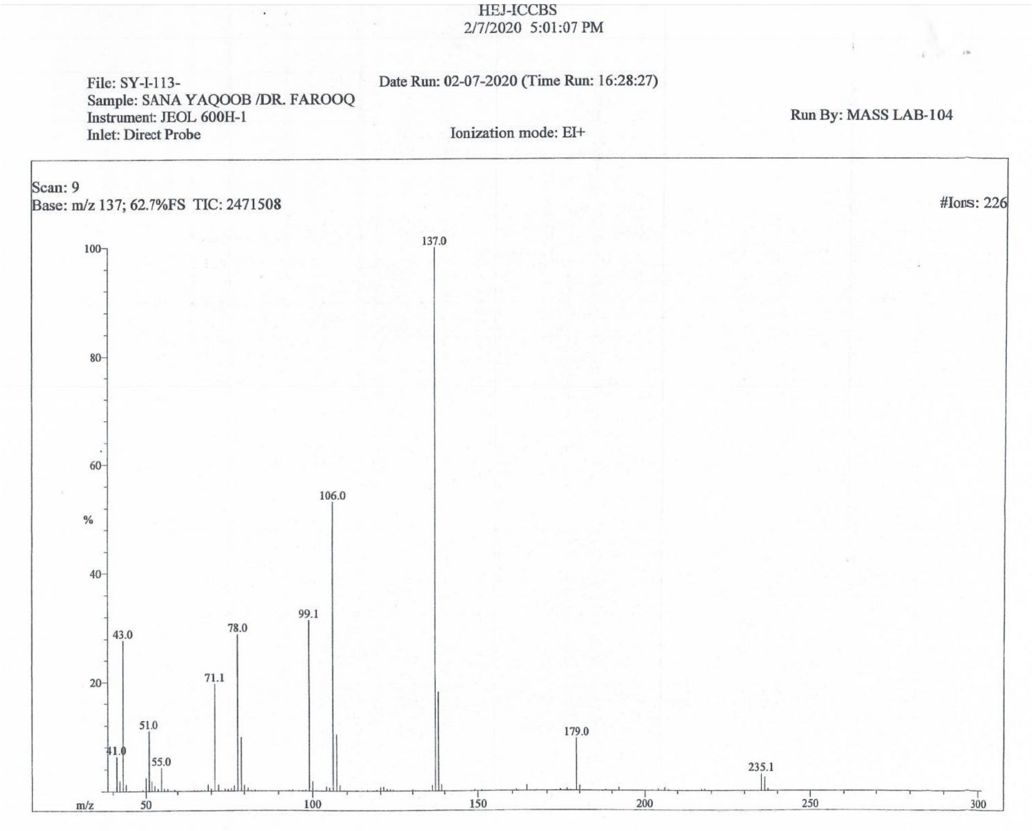

Figure S12. LR-MS (EI) spectrum of *N'*-Hexanoylpyridine-3-carbohydrazide (**5**)

File : D:\Data\sy-i-113_180220-c1.RAW
Full ms [49.500 - 750.500] - Range: 76.000 - 750.500
Scan No. 1 of 1

Mass	Relative Intensity	Theoretical Mass	Delta [ppm]	Delta [mmu]	RDB	Composition
76.0171	3.3	76.0160	13.9	1.1	1.0	$C_2H_4O_3$
77.0255	8.6	77.0239	20.9	1.6	0.5	$C_2H_5O_3$
78.0336	37.1	78.0317	24.7	1.9	0.0	$C_2H_6O_3$
79.0399	5.5	79.0382	21.5	1.7	0.0	$H_5O_2N_3$
80.0490	0.9					
81.0704	1.1					
82.0663	1.5					
83.0510	0.5					
83.0852	0.9					
84.0447	0.7					
87.0423	0.7	87.0446	-26.6	-2.3	1.5	$C_4H_7O_2$
90.0350	1.0					
91.0421	6.2	91.0395	28.5	2.6	0.5	$C_3H_7O_3$
92.0392	2.6					
93.0463	1.0					
94.0608	0.6					
95.0764	0.7					
96.0818	3.3					
97.0646	0.5					
97.0995	0.5					
98.0477	8.8					
99.0514	0.6					
99.0800	13.4					
100.0830	1.0					
103.0302	0.9					
104.0398	18.3					
105.0231	3.7					
105.0445	4.5	105.0426	18.0	1.9	1.0	$C_3H_7O_3N_1$
106.0281	39.8	106.0266	14.2	1.5	1.0	$C_3H_6O_4$
		106.0253	26.8	2.8	1.5	$C_1H_4O_3N_3$
107.0334	5.2	107.0331	2.6	0.3	1.0	$C_1H_5O_3N_3$
		107.0344	-9.9	-1.1	0.5	$C_3H_7O_4$
108.0389	0.5	108.0409	-18.5	-2.0	0.5	$C_1H_6O_3N_3$
111.0544	5.8					
111.0900	0.9					
112.0625	1.5					
116.0576	1.1	116.0586	-8.4	-1.0	2.0	$C_4H_8O_2N_2$
117.0583	1.3	117.0552	26.8	3.1	1.5	$C_5H_9O_3$
118.0643	3.0	118.0630	10.9	1.3	1.0	$C_5H_{10}O_3$
		118.0617	22.3	2.6	1.5	$C_3H_8O_2N_3$
119.0482	14.8	119.0457	21.0	2.5	1.5	$C_3H_7O_3N_2$
120.0551	4.9	120.0535	13.5	1.6	1.0	$C_3H_8O_3N_2$
121.0458	0.9	121.0487	-24.2	-2.9	1.0	$C_2H_7O_3N_3$
122.0475	2.7	122.0453	17.4	2.1	0.5	$C_3H_8O_4N_1$
		122.0440	28.4	3.5	1.0	$C_1H_6O_3N_4$
123.0342	5.3	123.0320	17.3	2.1	5.0	$C_6H_5O_2N_1$
123.0534	4.8	123.0532	2.1	0.3	0.0	$C_3H_9O_4N_1$
		123.0518	13.0	1.6	0.5	$C_1H_7O_3N_4$
124.0386	0.5	124.0399	-9.7	-1.2	4.5	$C_6H_6O_2N_1$
		124.0358	22.7	2.8	0.5	$C_1H_6O_4N_3$
124.0580	0.5	124.0596	-13.4	-1.7	0.0	$C_1H_8O_3N_4$
125.0723	2.0					
130.0654	0.6	130.0630	18.1	2.4	2.0	$C_6H_{10}O_3$
		130.0617	28.5	3.7	2.5	$C_4H_8O_2N_3$
130.1110	1.0					
131.0611	1.0	131.0582	21.8	2.9	2.0	$C_5H_9O_3N_1$
132.0525	1.2	132.0535	-7.6	-1.0	2.0	$C_4H_8O_3N_2$
133.0623	0.6	133.0613	7.4	1.0	1.5	$C_4H_9O_3N_2$
137.0575	29.9	137.0562	9.4	1.3	0.5	$C_3H_9O_4N_2$
		137.0603	-19.9	-2.7	4.5	$C_8H_9O_2$
138.0628	4.1	138.0641	-9.4	-1.3	0.0	$C_3H_{10}O_4N_2$
146.0675	1.4	146.0691	-11.5	-1.7	2.0	$C_5H_{10}O_3N_2$
148.0502	1.2	148.0484	12.3	1.8	2.0	$C_4H_8O_4N_2$
		148.0524	-14.9	-2.2	6.0	$C_9H_8O_2$
149.0226	1.8	149.0225	0.5	0.1	7.0	$C_6H_3O_2N_3$
		149.0239	-8.5	-1.3	6.5	$C_8H_5O_3$
160.0528	0.8	160.0524	2.2	0.3	7.0	$C_{10}H_8O_2$

Mass	Relative Intensity	Theoretical Mass	Delta [ppm]	Delta [mmu]	RDB	Composition
		160.0484	27.3	4.4	3.0	$C_5H_8O_4N_2$
161.0592	100.0	161.0603	-6.5	-1.0	6.5	$C_{10}H_9O_2$
		161.0562	18.5	3.0	2.5	$C_5H_9O_4N_2$
162.0612	8.5	162.0641	-17.3	-2.8	2.0	$C_5H_{10}O_4N_2$
163.0637	0.8	163.0633	2.3	0.4	6.0	$C_9H_9O_2N_1$
		163.0593	27.0	4.4	2.0	$C_4H_9O_4N_3$
167.0346	0.6	167.0344	1.2	0.2	5.5	$C_8H_7O_4$
		167.0331	9.2	1.5	6.0	$C_6H_5O_3N_3$
173.0573	0.7	173.0562	6.4	1.1	3.5	$C_6H_9O_4N_2$
		173.0603	-16.9	-2.9	7.5	$C_{11}H_9O_2$
174.0656	83.4	174.0641	8.8	1.5	3.0	$C_6H_{10}O_4N_2$
		174.0681	-14.3	-2.5	7.0	$C_{11}H_{10}O_2$
175.0724	17.6	175.0719	2.9	0.5	2.5	$C_6H_{11}O_4N_2$
		175.0759	-20.1	-3.5	6.5	$C_{11}H_{11}O_2$
176.0773	1.8	176.0797	-13.7	-2.4	2.0	$C_6H_{12}O_4N_2$
179.0685	2.3	179.0695	-5.4	-1.0	6.0	$C_8H_9O_2N_3$
		179.0708	-12.9	-2.3	5.5	$C_{10}H_{11}O_3$
188.0835	22.2	188.0837	-1.3	-0.2	7.0	$C_{12}H_{12}O_2$
		188.0797	20.1	3.8	3.0	$C_7H_{12}O_4N_2$
189.0873	3.8	189.0875	-1.1	-0.2	2.5	$C_7H_{13}O_4N_2$
		189.0916	-22.4	-4.2	6.5	$C_{12}H_{13}O_2$
200.0816	1.1	200.0797	9.2	1.8	4.0	$C_8H_{12}O_4N_2$
		200.0837	-10.9	-2.2	8.0	$C_{13}H_{12}O_2$
202.0963	1.4	202.0954	4.6	0.9	3.0	$C_8H_{14}O_4N_2$
		202.0994	-15.3	-3.1	7.0	$C_{13}H_{14}O_2$
215.1059	0.9	215.1072	-6.1	-1.3	7.5	$C_{14}H_{15}O_2$
		215.1032	12.6	2.7	3.5	$C_9H_{15}O_4N_2$
216.1138	2.4	216.1150	-5.6	-1.2	7.0	$C_{14}H_{16}O_2$
		216.1110	13.0	2.8	3.0	$C_9H_{16}O_4N_2$
217.1215	10.4	217.1229	-6.1	-1.3	6.5	$C_{14}H_{17}O_2$
		217.1188	12.4	2.7	2.5	$C_9H_{17}O_4N_2$
218.1261	2.0	218.1267	-2.8	-0.6	2.0	$C_9H_{18}O_4N_2$
		218.1307	-21.2	-4.6	6.0	$C_{14}H_{18}O_2$
235.1320	0.6	235.1321	-0.5	-0.1	6.0	$C_{12}H_{17}O_2N_3$
		235.1334	-6.2	-1.5	5.5	$C_{14}H_{19}O_3$

Figure S13. HR-MS (EI) spectral data of *N*'-Hexanoylpyridine-3-carbohydrazide (**5**)

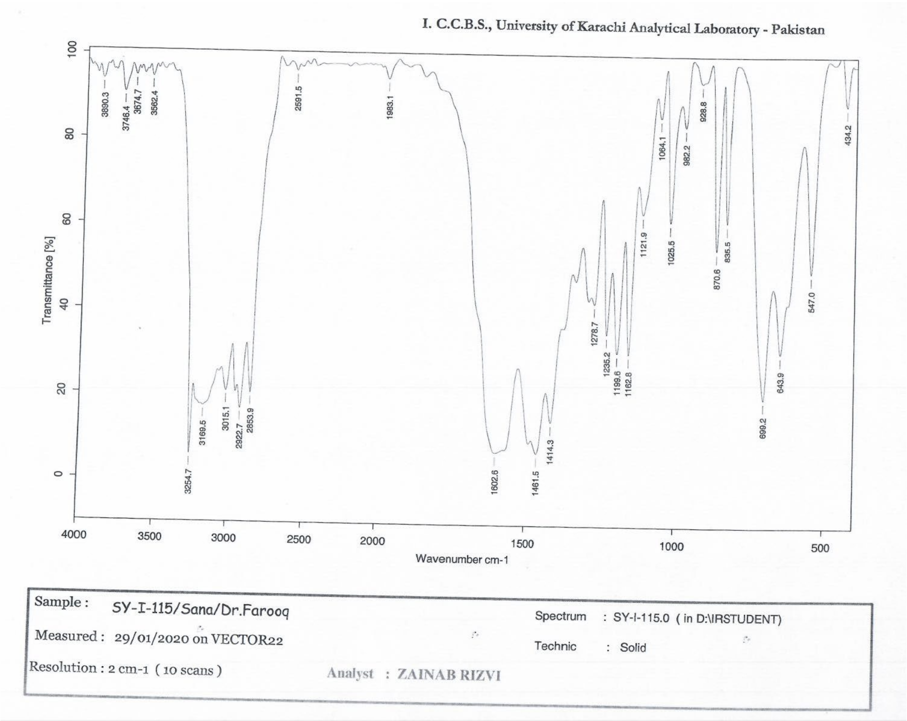

Figure S14. IR spectrum of *N*'-Octanoylpyridine-3-carbohydrazide (**6**)

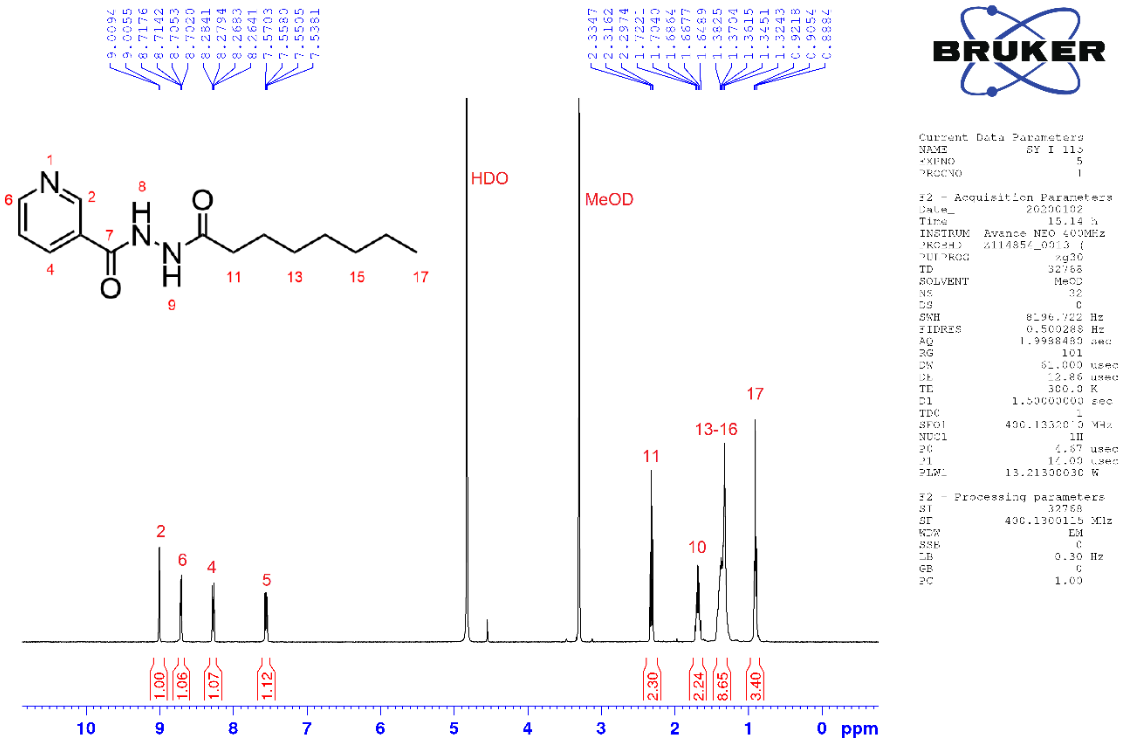

Figure S15. ^{1}H NMR spectrum of *N*'-Octanoylpyridine-3-carbohydrazide (**6**)

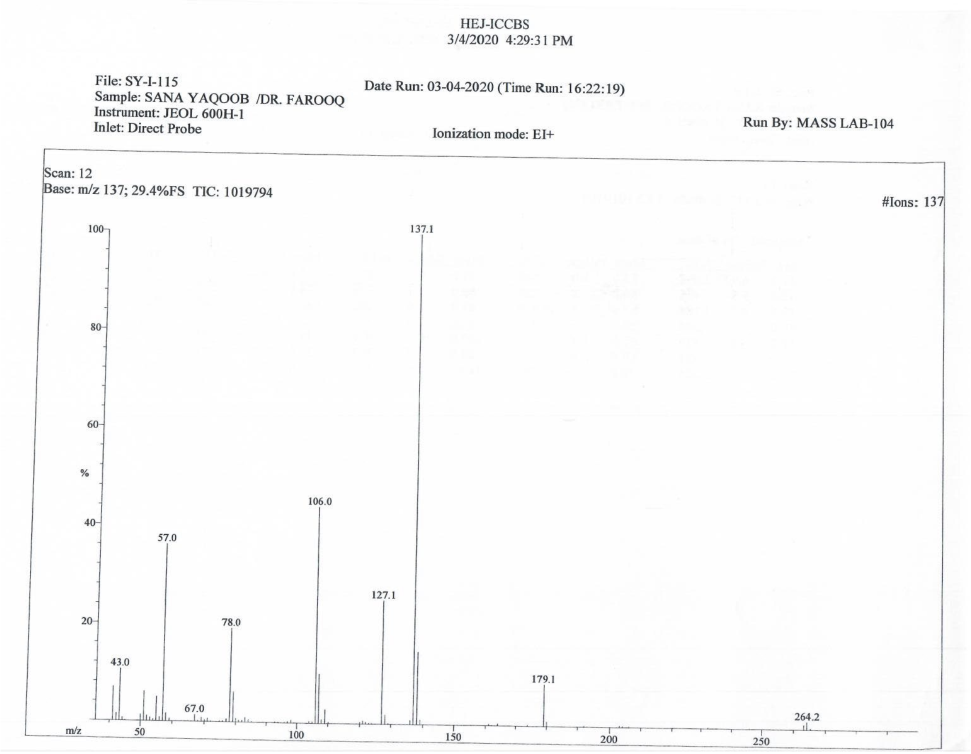

Figure S16. LR-MS (EI) spectrum of *N*'-Octanoylpyridine-3-carbohydrazide (**6**)

File : D:\Data\sy-i-115-160320-c3.RAW
Full ms [49.500 - 700.500] - Range: 67.000 - 700.500
Scan No. 1 of 1

Mass	Relative Intensity	Theoretical Mass	Delta [ppm]	Delta [mmu]	RDB	Composition
67.0558	2.1	67.0548	16.0	1.1	2.5	$C_5 H_7$
69.0705	1.5	69.0704	1.1	0.1	1.5	$C_5 H_9$
71.0866	0.7	71.0861	7.3	0.5	0.5	$C_5 H_{11}$
76.0185	1.0	76.0187	-2.8	-0.2	5.5	$C_5 H_2 N_1$
77.0264	2.8	77.0265	-2.3	-0.2	5.0	$C_5 H_3 N_1$
78.0347	24.8	78.0344	4.5	0.4	4.5	$C_5 H_4 N_1$
79.0416	6.4	79.0422	-7.1	-0.6	4.0	$C_5 H_5 N_1$
80.0486	0.9	80.0500	-18.0	-1.4	3.5	$C_5 H_6 N_1$
81.0702	0.6	81.0704	-3.0	-0.2	2.5	$C_6 H_9$
82.0669	1.8	82.0657	15.1	1.2	2.5	$C_5 H_8 N_1$
83.0504	0.6	83.0497	8.8	0.7	2.5	$C_5 H_7 O_1$
		83.0483	25.0	2.1	3.0	$C_3 H_5 N_3$
83.0834	1.2					
84.0571	0.8	84.0575	-5.1	-0.4	2.0	$C_5 H_8 O_1$
		84.0562	10.9	0.9	2.5	$C_3 H_6 N_3$
91.0422	0.7	91.0422	-0.0	-0.0	5.0	$C_6 H_5 N_1$
		91.0395	29.4	2.7	0.5	$C_3 H_7 O_3$
92.0383	0.5	92.0374	9.1	0.8	5.0	$C_5 H_4 N_2$
96.0812	1.0	96.0813	-1.6	-0.2	2.5	$C_6 H_{10} N_1$
97.0896	0.7	97.0891	4.9	0.5	2.0	$C_6 H_{11} N_1$
98.0715	0.9	98.0718	-2.8	-0.3	2.5	$C_4 H_8 N_3$
		98.0732	-16.5	-1.6	2.0	$C_6 H_{10} O_1$
104.0378	4.5	104.0374	3.2	0.3	6.0	$C_6 H_4 N_2$
		104.0348	29.0	3.0	1.5	$C_3 H_6 O_3 N_1$
105.0438	1.0	105.0426	11.9	1.3	1.0	$C_3 H_7 O_3 N_1$
		105.0453	-13.6	-1.4	5.5	$C_6 H_5 N_2$
106.0283	46.1	106.0293	-9.7	-1.0	5.5	$C_6 H_4 O_1 N_1$
		106.0266	15.6	1.7	1.0	$C_3 H_6 O_4$
		106.0253	28.3	3.0	1.5	$C_1 H_4 O_3 N_3$
107.0356	9.3	107.0344	11.2	1.2	0.5	$C_3 H_7 O_4$
		107.0371	-13.8	-1.5	5.0	$C_6 H_5 O_1 N_1$
		107.0331	23.8	2.5	1.0	$C_1 H_5 O_3 N_3$
108.0399	0.8	108.0409	-9.0	-1.0	0.5	$C_1 H_6 O_3 N_3$
		108.0423	-21.5	-2.3	0.0	$C_3 H_8 O_4$
109.1009	2.8	109.1017	-7.3	-0.8	2.5	$C_8 H_{13}$
119.0479	2.1	119.0483	-3.6	-0.4	6.0	$C_6 H_5 N_3$
		119.0497	-14.9	-1.8	5.5	$C_8 H_7 O_1$
		119.0457	18.9	2.2	1.5	$C_3 H_7 O_3 N_2$
120.0546	1.5	120.0535	8.9	1.1	1.0	$C_3 H_8 O_3 N_2$
		120.0562	-13.4	-1.6	5.5	$C_6 H_6 N_3$
		120.0575	-24.6	-3.0	5.0	$C_8 H_8 O_1$
121.0405	0.8	121.0402	2.9	0.4	5.5	$C_6 H_5 O_1 N_2$
		121.0375	25.1	3.0	1.0	$C_3 H_7 O_4 N_1$
122.0476	1.0	122.0480	-3.0	-0.4	5.0	$C_6 H_6 O_1 N_2$
		122.0453	18.9	2.3	0.5	$C_3 H_8 O_4 N_1$
123.0522	0.8	123.0532	-8.2	-1.0	0.0	$C_3 H_9 O_4 N_1$
		123.0558	-30.0	-3.7	4.5	$C_6 H_7 O_1 N_2$
127.1132	20.2	127.1123	6.8	0.9	1.5	$C_8 H_{15} O_1$
		127.1109	17.3	2.2	2.0	$C_6 H_{13} N_3$
128.1153	1.7	128.1188	-26.8	-3.4	1.5	$C_6 H_{14} N_3$
136.0516	0.7	136.0511	4.0	0.6	5.5	$C_6 H_6 O_1 N_3$
		136.0524	-5.8	-0.8	5.0	$C_8 H_8 O_2$
		136.0484	23.7	3.2	1.0	$C_3 H_8 O_4 N_2$
137.0585	100.0	137.0589	-2.7	-0.4	5.0	$C_6 H_7 O_1 N_3$
		137.0603	-12.5	-1.7	4.5	$C_8 H_9 O_2$
		137.0562	16.9	2.3	0.5	$C_3 H_9 O_4 N_2$
138.0644	12.0	138.0641	2.6	0.4	0.0	$C_3 H_{10} O_4 N_2$
		138.0667	-16.9	-2.3	4.5	$C_6 H_8 O_1 N_3$
		138.0681	-26.6	-3.7	4.0	$C_8 H_{10} O_2$
139.0667	0.9	139.0633	24.4	3.4	4.0	$C_7 H_9 O_2 N_1$
161.0591	13.5	161.0589	1.3	0.2	7.0	$C_8 H_7 O_1 N_3$
		161.0603	-7.0	-1.1	6.5	$C_{10} H_9 O_2$
		161.0562	18.0	2.9	2.5	$C_5 H_9 O_4 N_2$
162.0620	1.5	162.0641	-12.7	-2.1	2.0	$C_5 H_{10} O_4 N_2$
		162.0667	-29.2	-4.7	6.5	$C_8 H_8 O_1 N_3$
174.0662	11.5	174.0667	-3.4	-0.6	7.5	$C_9 H_8 O_1 N_3$
		174.0681	-11.1	-1.9	7.0	$C_{11} H_{10} O_2$

Mass	Relative Intensity	Theoretical Mass	Delta [ppm]	Delta [mmu]	RDB	Composition
		174.0641	12.0	2.1	3.0	$C_6 H_{10} O_4 N_2$
175.0725	2.6	175.0719	3.6	0.6	2.5	$C_6 H_{11} O_4 N_2$
		175.0746	-11.7	-2.1	7.0	$C_9 H_9 O_1 N_3$
		175.0759	-19.4	-3.4	6.5	$C_{11} H_{11} O_2$
179.0698	6.2	179.0695	2.1	0.4	6.0	$C_8 H_9 O_2 N_3$
		179.0708	-5.4	-1.0	5.5	$C_{10} H_{11} O_3$
		179.0668	17.0	3.1	1.5	$C_5 H_{11} O_5 N_2$
		179.0735	-20.4	-3.7	10.0	$C_{13} H_9 N_1$
180.0708	0.7	180.0687	11.6	2.1	10.0	$C_{12} H_8 N_2$
		180.0746	-21.0	-3.8	1.0	$C_5 H_{12} O_5 N_2$
		180.0661	26.5	4.8	5.5	$C_9 H_{10} O_3 N_1$
188.0819	1.3	188.0824	-2.6	-0.5	7.5	$C_{10} H_{10} O_1 N_3$
		188.0837	-9.7	-1.8	7.0	$C_{12} H_{12} O_2$
		188.0797	11.7	2.2	3.0	$C_7 H_{12} O_4 N_2$
202.0978	2.9	202.0980	-1.1	-0.2	7.5	$C_{11} H_{12} O_1 N_3$
		202.0994	-7.7	-1.6	7.0	$C_{13} H_{14} O_2$
		202.0954	12.2	2.5	3.0	$C_8 H_{14} O_4 N_2$
203.1049	4.8	203.1059	-5.0	-1.0	7.0	$C_{11} H_{13} O_1 N_3$
		203.1032	8.2	1.7	2.5	$C_8 H_{15} O_4 N_2$
		203.1072	-11.6	-2.3	6.5	$C_{13} H_{15} O_2$
204.1101	0.8	204.1110	-4.5	-0.9	2.0	$C_8 H_{16} O_4 N_2$
		204.1137	-17.6	-3.6	6.5	$C_{11} H_{14} O_1 N_3$
		204.1150	-24.2	-4.9	6.0	$C_{13} H_{16} O_2$
216.1145	4.3	216.1150	-2.7	-0.6	7.0	$C_{14} H_{16} O_2$
		216.1137	3.6	0.8	7.5	$C_{12} H_{14} O_1 N_3$
		216.1110	16.0	3.4	3.0	$C_9 H_{16} O_4 N_2$
217.1200	1.4	217.1188	5.4	1.2	2.5	$C_9 H_{17} O_4 N_2$
		217.1215	-6.9	-1.5	7.0	$C_{12} H_{15} O_1 N_3$
		217.1229	-13.1	-2.9	6.5	$C_{14} H_{17} O_2$
230.1306	0.6	230.1307	-0.2	-0.1	7.0	$C_{15} H_{18} O_2$
		230.1293	5.6	1.3	7.5	$C_{13} H_{16} O_1 N_3$
		230.1267	17.2	4.0	3.0	$C_{10} H_{18} O_4 N_2$
245.1537	0.8	245.1542	-1.8	-0.4	6.5	$C_{16} H_{21} O_2$
		245.1528	3.7	0.9	7.0	$C_{14} H_{19} O_1 N_3$
		245.1501	14.6	3.6	2.5	$C_{11} H_{21} O_4 N_2$
263.1636	0.6	263.1634	0.9	0.2	6.0	$C_{14} H_{21} O_2 N_3$
		263.1647	-4.2	-1.1	5.5	$C_{16} H_{23} O_3$
		263.1607	11.1	2.9	1.5	$C_{11} H_{23} O_5 N_2$
		263.1674	-14.4	-3.8	10.0	$C_{19} H_{21} N_1$

Figure S17. HR-MS (EI) spectral data of *N*'-Octanoylpyridine-3-carbohydrazide (**6**)

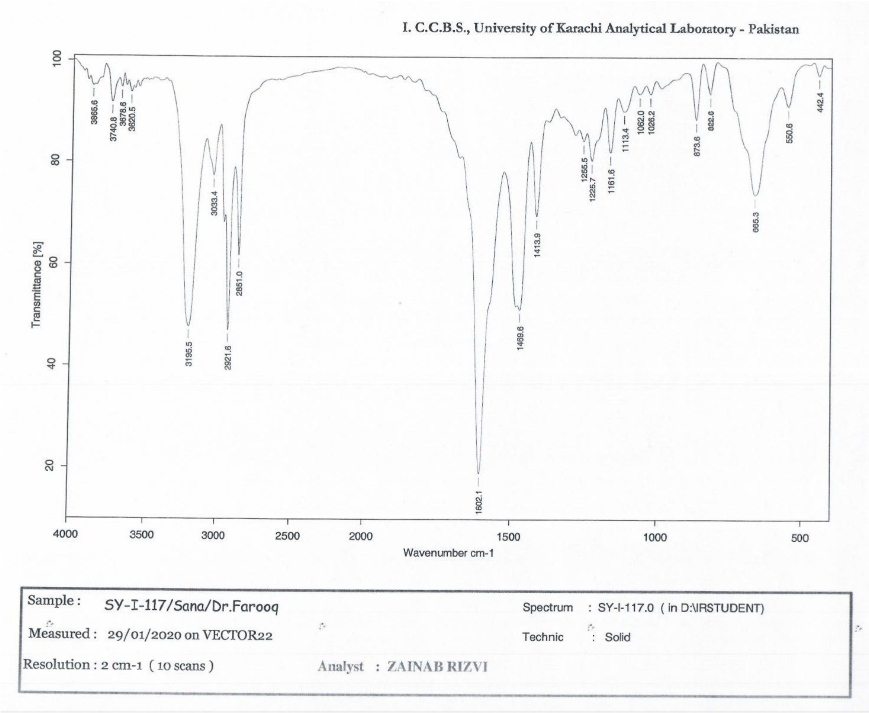

Figure S18. IR spectrum of *N'*-Decanoylpyridine-3-carbohydrazide (**7**)

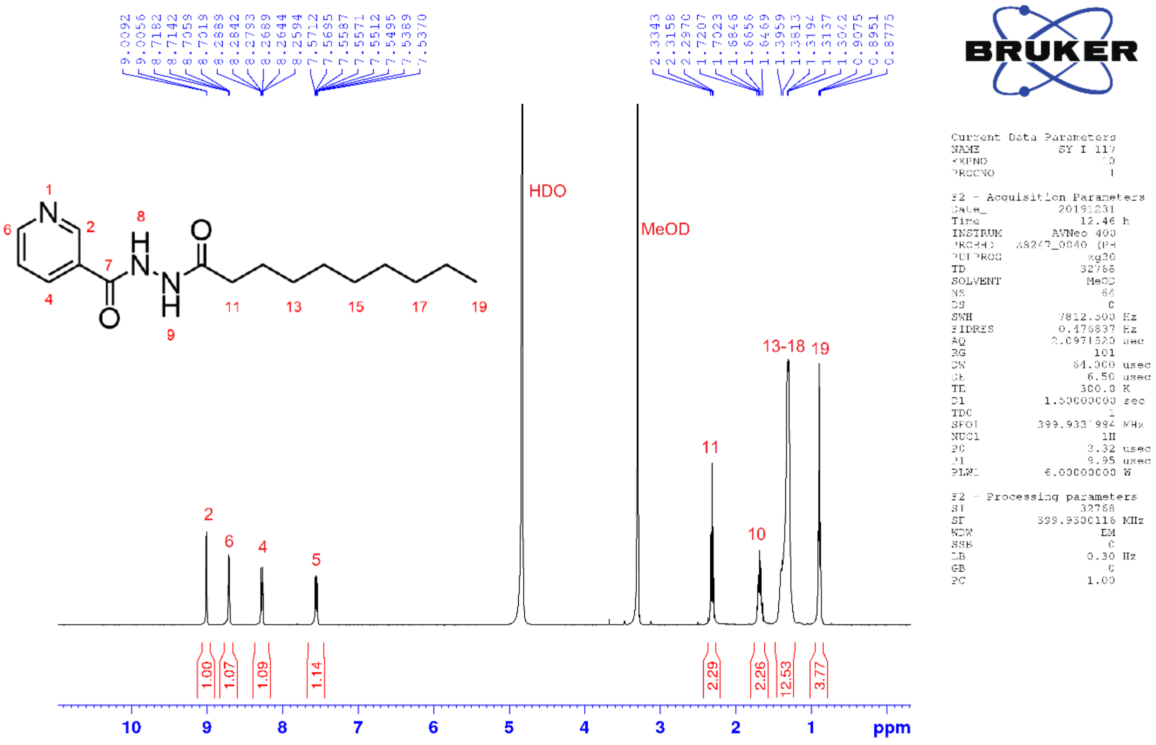

Figure S19. ^{1}H NMR spectrum of *N'*-Decanoylpyridine-3-carbohydrazide (**7**)

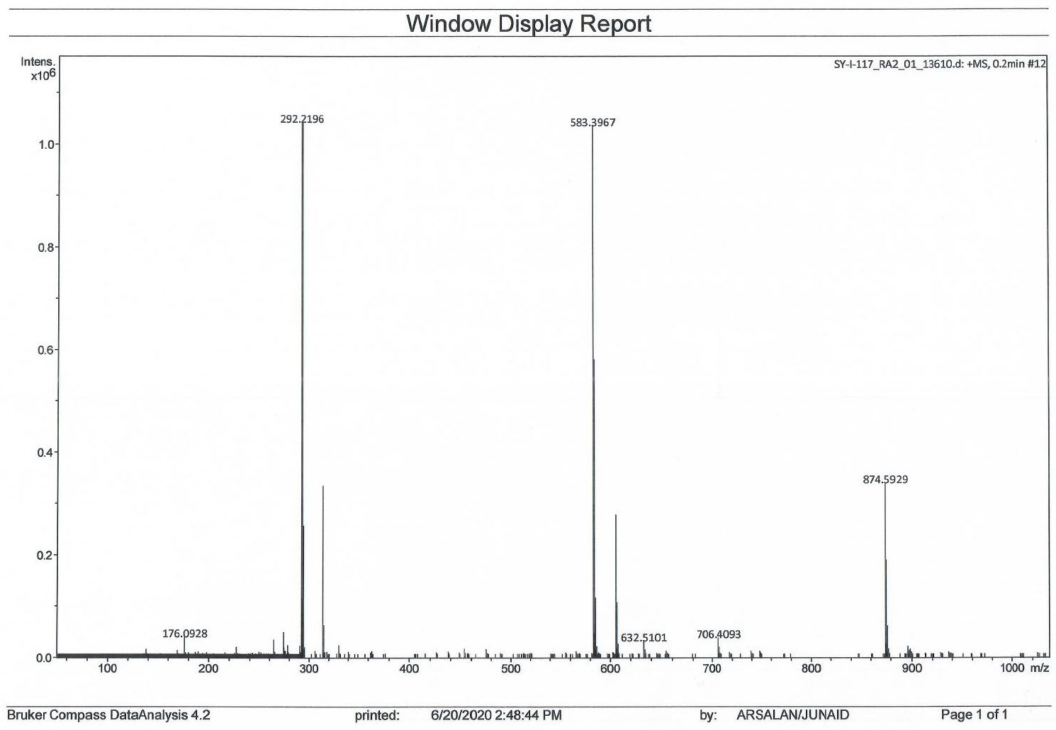

Figure S20. LR-MS (EI) spectrum of *N*'-Decanoylpyridine-3-carbohydrazide (**7**)

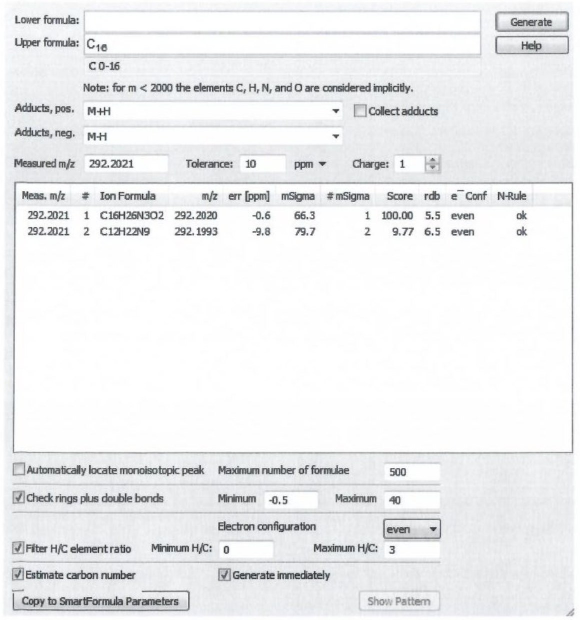

Figure S21. HR-MS (EI) spectral data of *N*'-Decanoylpyridine-3-carbohydrazide (**7**)

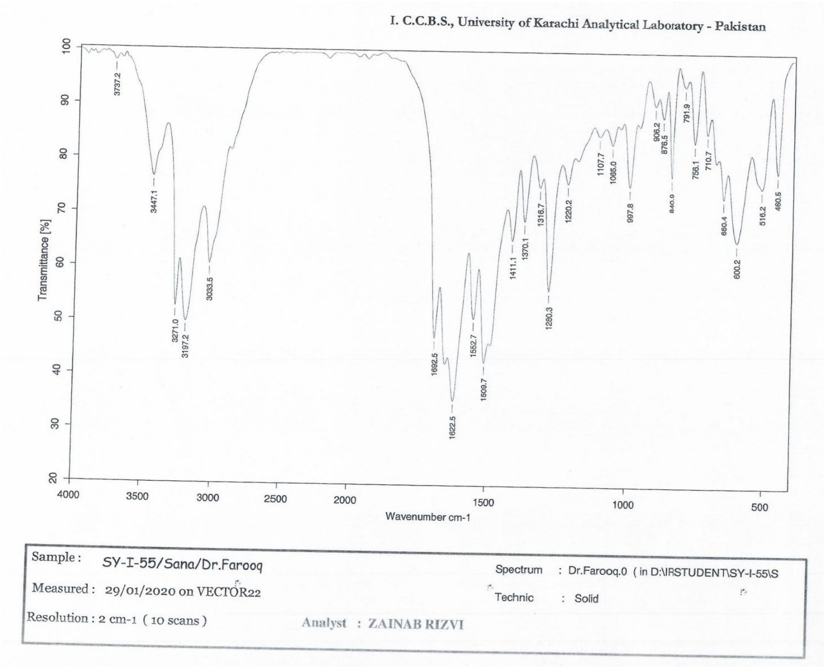

Figure S22. IR spectrum of *N*'-Acetylpyridine-4-carbohydrazide (**8**)

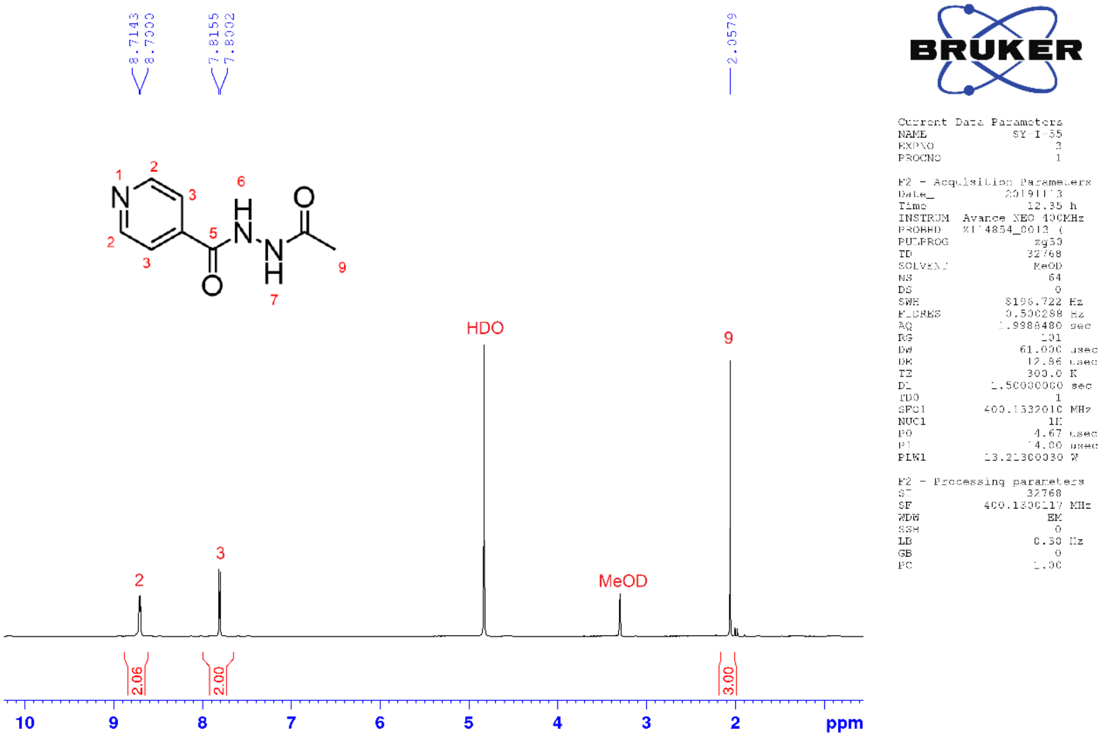

Figure S23. ^{1}H NMR spectrum of *N*'-Acetylpyridine-4-carbohydrazide (**8**)

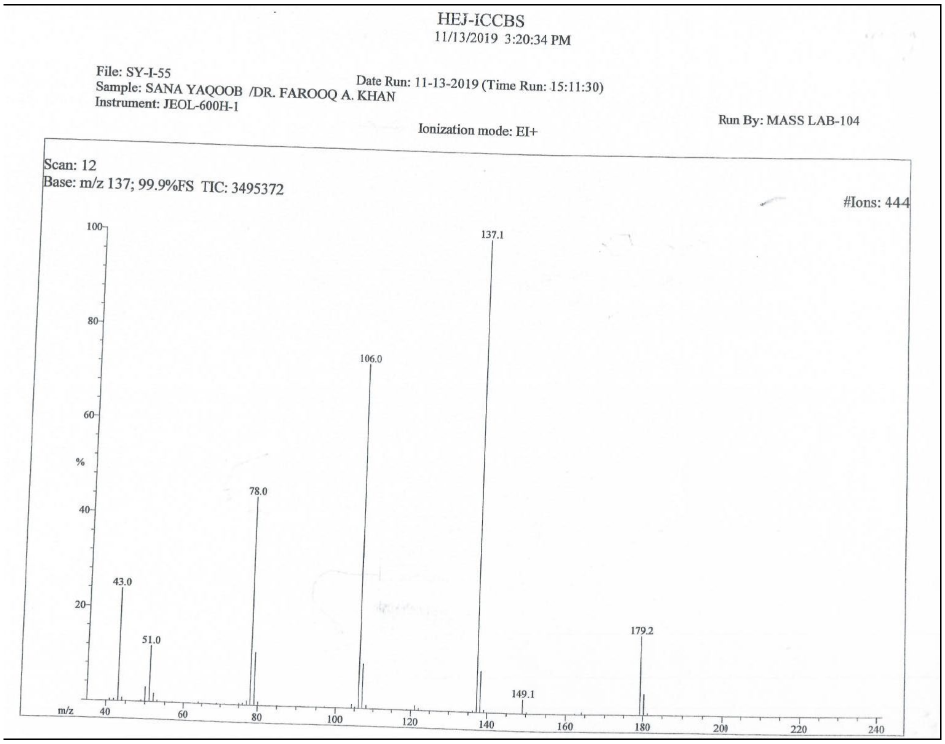

Figure S24. LR-MS (EI) spectrum of *N*'-Acetylpyridine-4-carbohydrazide (**8**)

File : D:\Data\sy-i-55-140220-c8.RAW
Full ms [49.500 - 750.500] - Range: 62.000 - 750.500
Scan No. 1 of 1

Mass	Relative Intensity	Theoretical Mass	Delta [ppm]	Delta [mmu]	RDB	Composition
62.0255	2.5	62.0242	21.4	1.3	0.5	$C_1 H_4 O_2 N_1$
63.0314	4.2	63.0320	-9.2	-0.6	0.0	$C_1 H_5 O_2 N_1$
64.0364	5.8					
65.0337	2.0					
76.0163	4.6	76.0160	3.2	0.2	1.0	$C_2 H_4 O_3$
		76.0147	20.9	1.6	1.5	$H_2 O_2 N_3$
77.0241	10.6	77.0239	2.6	0.2	0.5	$C_2 H_5 O_3$
		77.0225	20.0	1.5	1.0	$H_3 O_2 N_3$
78.0322	73.5	78.0317	5.9	0.5	0.0	$C_2 H_6 O_3$
		78.0304	23.1	1.8	0.5	$H_4 O_2 N_3$
79.0391	11.6	79.0382	11.6	0.9	0.0	$H_5 O_2 N_3$
91.0410	21.3	91.0395	16.4	1.5	0.5	$C_3 H_7 O_3$
92.0424	2.4					
104.0418	22.4					
105.0292	3.3	105.0300	-7.9	-0.8	1.5	$C_2 H_5 O_3 N_2$
105.0573	7.9	105.0552	20.2	2.1	0.5	$C_4 H_9 O_3$
106.0283	73.6	106.0266	16.2	1.7	1.0	$C_3 H_6 O_4$
		106.0253	28.9	3.1	1.5	$C_1 H_4 O_3 N_3$
107.0328	6.7	107.0331	-2.9	-0.3	1.0	$C_1 H_5 O_3 N_3$
		107.0344	-15.5	-1.7	0.5	$C_3 H_7 O_4$
119.0472	7.5	119.0457	13.3	1.6	1.5	$C_3 H_7 O_3 N_2$
120.0359	1.6					
122.0480	2.5	122.0453	22.2	2.7	0.5	$C_3 H_8 O_4 N_1$
123.0313	14.8	123.0320	-5.8	-0.7	5.0	$C_6 H_5 O_2 N_1$
		123.0280	26.9	3.3	1.0	$C_1 H_5 O_4 N_3$
124.0350	1.1	124.0358	-6.6	-0.8	0.5	$C_1 H_6 O_4 N_3$
132.0460	2.4	132.0423	28.3	3.7	2.0	$C_5 H_8 O_4$
137.0588	49.2	137.0603	-10.3	-1.4	4.5	$C_8 H_9 O_2$
		137.0562	19.0	2.6	0.5	$C_3 H_9 O_4 N_2$
138.0610	3.7	138.0641	-22.1	-3.0	0.0	$C_3 H_{10} O_4 N_2$
149.0474	1.6	149.0477	-1.8	-0.3	6.0	$C_8 H_7 O_2 N_1$
		149.0437	25.2	3.8	2.0	$C_3 H_7 O_4 N_3$
161.0578	100.0	161.0562	10.0	1.6	2.5	$C_5 H_9 O_4 N_2$
		161.0603	-15.0	-2.4	6.5	$C_{10} H_9 O_2$
162.0611	8.7	162.0641	-18.1	-2.9	2.0	$C_5 H_{10} O_4 N_2$
163.0426	1.5	163.0395	19.2	3.1	6.5	$C_9 H_7 O_3$
		163.0382	27.4	4.5	7.0	$C_7 H_5 O_2 N_3$
179.0696	5.2	179.0695	0.9	0.2	6.0	$C_8 H_9 O_2 N_3$
		179.0708	-6.6	-1.2	5.5	$C_{10} H_{11} O_3$

Figure S25. HR-MS (EI) spectral data of *N*'-Acetylpyridine-4-carbohydrazide (**8**)

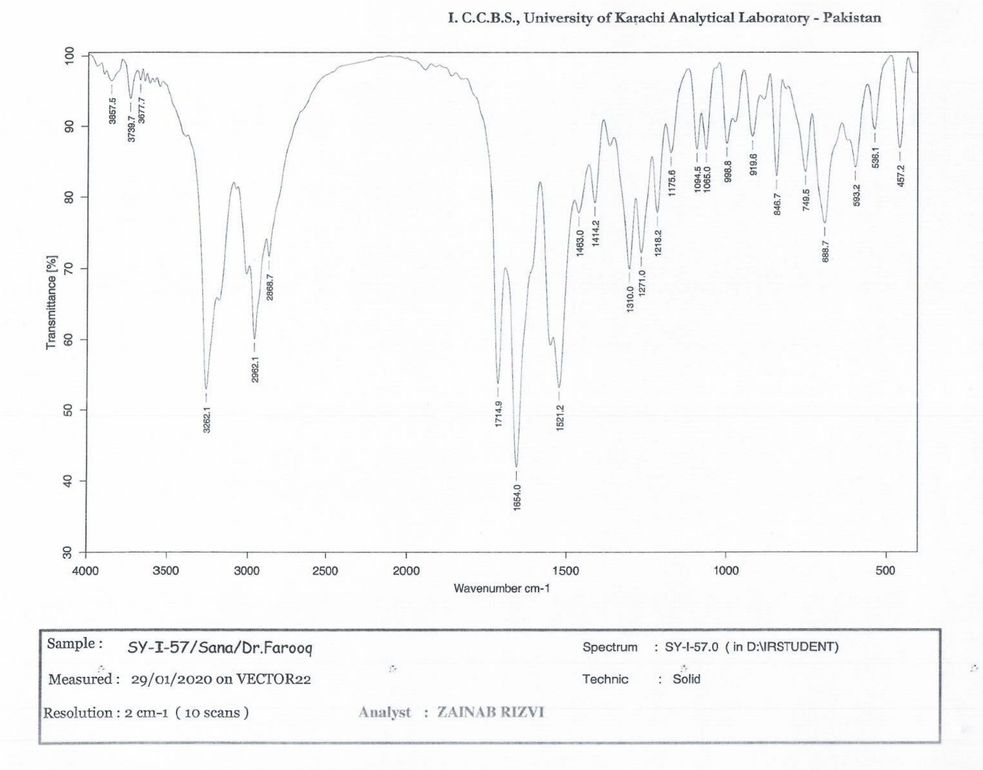

Figure S26. IR spectrum of *N*'-Butyrylpyridine-4-carbohydrazide (**9**)

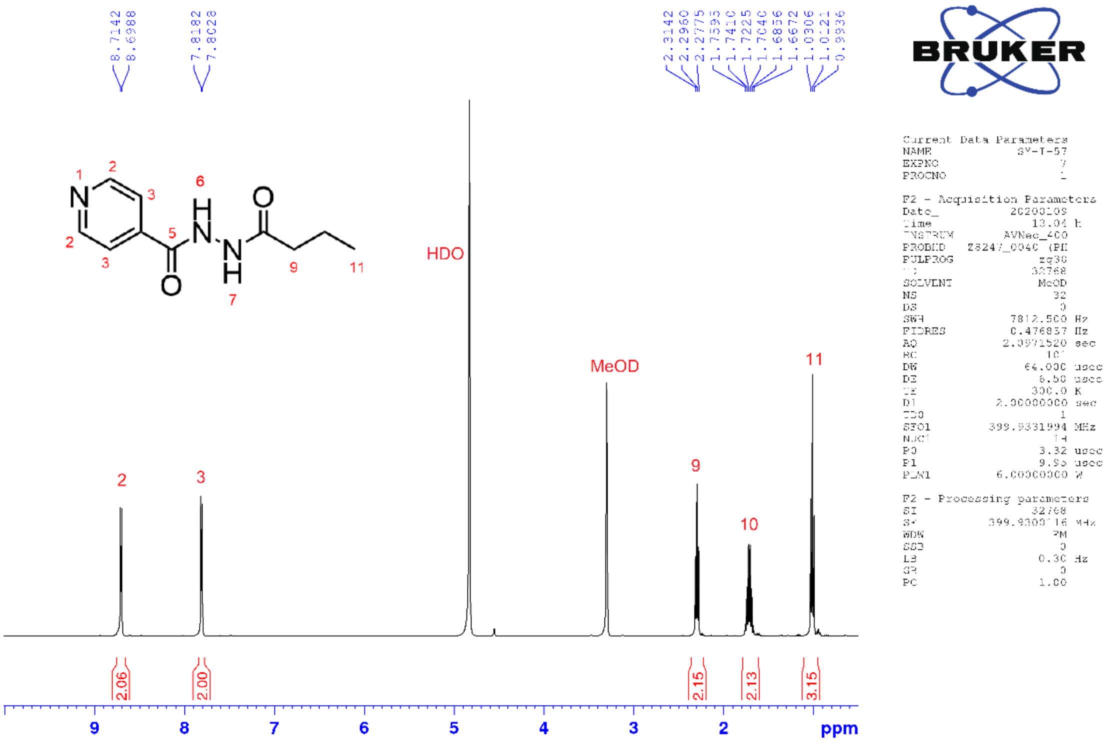

Figure S27. ^{1}H NMR spectrum of *N*'-Butyrylpyridine-4-carbohydrazide (**9**)

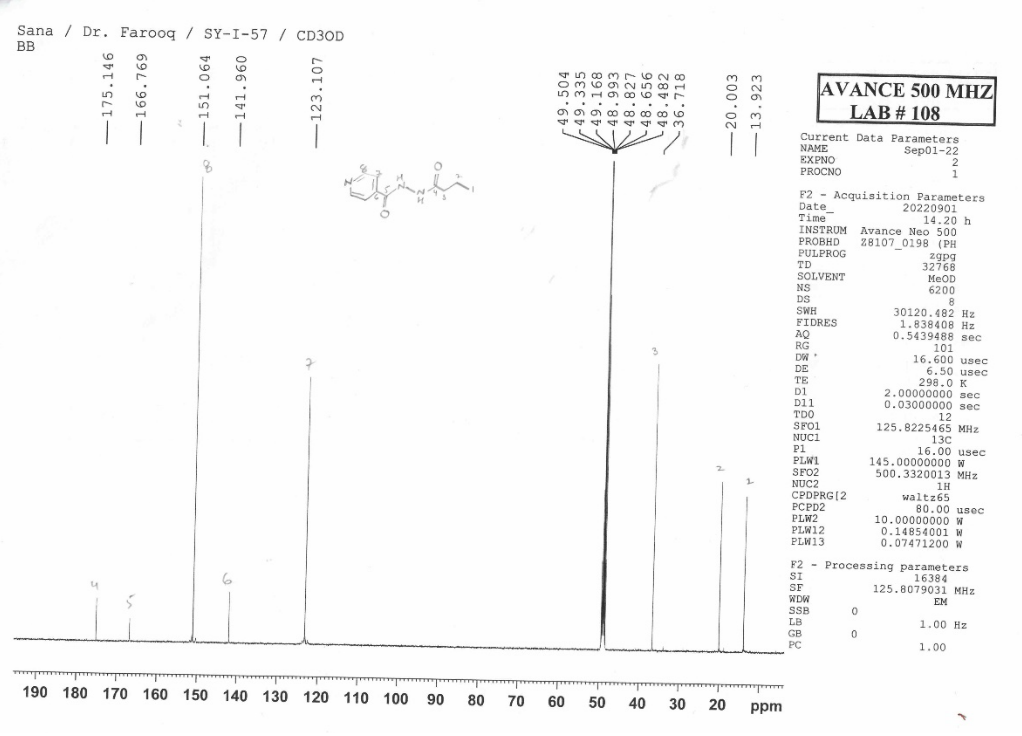

Figure S28. ^{13}C NMR spectrum of *N*'-Butyrylpyridine-4-carbohydrazide (**9**)

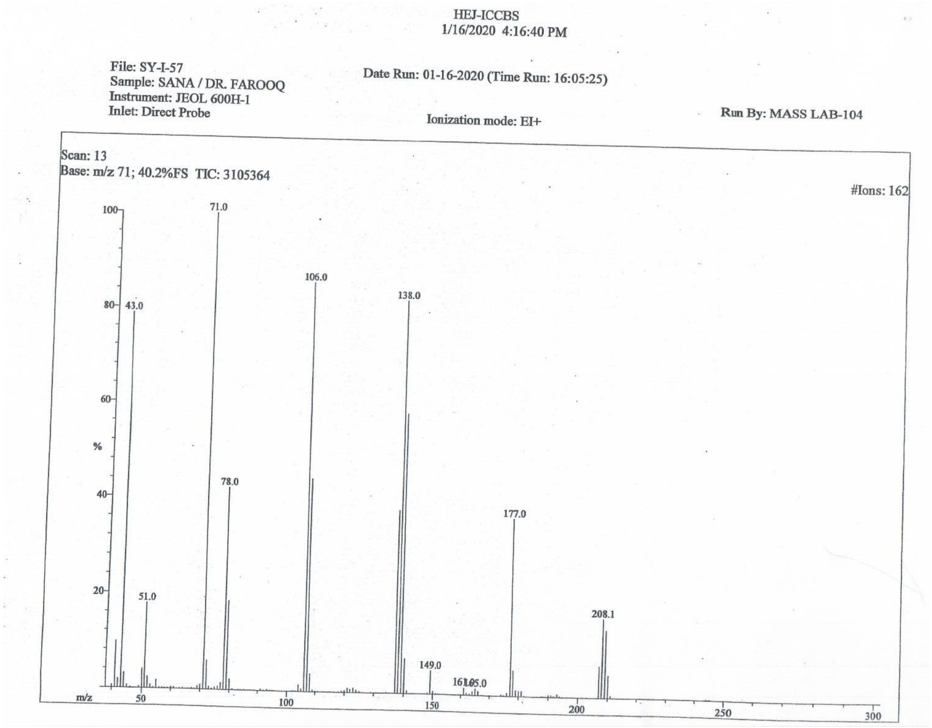

Figure S29. LR-MS (EI) spectrum of *N*'-Butyrylpyridine-4-carbohydrazide (**9**)

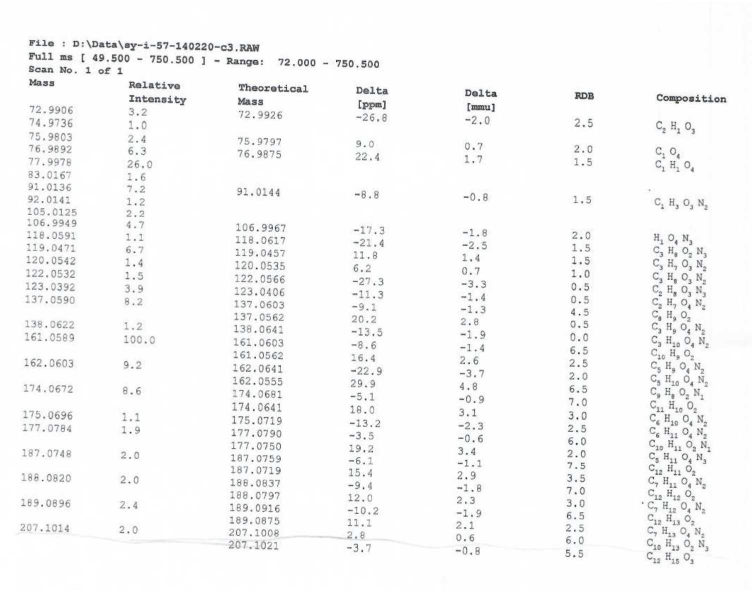

File : D:\Data\sy-i-57-140220-c3.RAW
Full ms [49.500 - 750.500] - Range: 72.000 - 750.500
Scan No. 1 of 1

Mass	Relative Intensity	Theoretical Mass	Delta [ppm]	Delta [mmu]	RDB	Composition
72.9906	3.2	72.9926	-26.8	-2.0	2.5	$C_2H_1O_3$
74.9736	1.0					
75.9803	2.4	75.9797	9.0	0.7	2.0	C_1O_4
76.9892	6.3	76.9875	22.4	1.7	1.5	$C_1H_1O_4$
77.9978	26.0					
83.0167	1.6					
91.0136	7.2	91.0144	-8.8	-0.8	1.5	$C_1H_3O_3N_2$
92.0141	1.2					
105.0125	2.2					
106.9949	4.7	106.9967	-17.3	-1.8	2.0	$H_1O_4N_3$
118.0591	1.1	118.0617	-21.4	-2.5	1.5	$C_3H_8O_2N_3$
119.0471	6.7	119.0457	11.8	1.4	1.5	$C_3H_7O_3N_2$
120.0542	1.4	120.0535	6.2	0.7	1.0	$C_3H_8O_3N_2$
122.0532	1.5	122.0566	-27.3	-3.3	0.5	$C_2H_8O_3N_3$
123.0392	3.9	123.0406	-11.3	-1.4	0.5	$C_2H_7O_4N_2$
137.0590	8.2	137.0603	-9.1	-1.3	4.5	$C_8H_9O_2$
		137.0562	20.2	2.8	0.5	$C_3H_9O_4N_2$
138.0622	1.2	138.0641	-13.5	-1.9	0.0	$C_3H_{10}O_4N_2$
161.0589	100.0	161.0603	-8.6	-1.4	6.5	$C_{10}H_9O_2$
		161.0562	16.4	2.6	2.5	$C_5H_9O_4N_2$
162.0603	9.2	162.0641	-22.9	-3.7	2.0	$C_5H_{10}O_4N_2$
		162.0555	29.9	4.8	6.5	$C_9H_8O_2N_1$
174.0672	8.6	174.0681	-5.1	-0.9	7.0	$C_{11}H_{10}O_2$
		174.0641	18.0	3.1	3.0	$C_6H_{10}O_4N_2$
175.0696	1.1	175.0719	-13.2	-2.3	2.5	$C_6H_{11}O_4N_2$
177.0784	1.9	177.0790	-3.5	-0.6	6.0	$C_{10}H_{11}O_2N_1$
		177.0750	19.2	3.4	2.0	$C_5H_{11}O_4N_3$
187.0748	2.0	187.0759	-6.1	-1.1	7.5	$C_{12}H_{11}O_2$
		187.0719	15.4	2.9	3.5	$C_7H_{11}O_4N_2$
188.0820	2.0	188.0837	-9.4	-1.8	7.0	$C_{12}H_{12}O_2$
		188.0797	12.0	2.3	3.0	$C_7H_{12}O_4N_2$
189.0896	2.4	189.0916	-10.2	-1.9	6.5	$C_{12}H_{13}O_2$
		189.0875	11.1	2.1	2.5	$C_7H_{13}O_4N_2$
207.1014	2.0	207.1008	2.8	0.6	6.0	$C_{10}H_{13}O_2N_3$
		207.1021	-3.7	-0.8	5.5	$C_{12}H_{15}O_3$

Figure S30. HR-MS (EI) spectral data of *N*'-Butyrylpyridine-4-carbohydrazide (**9**)

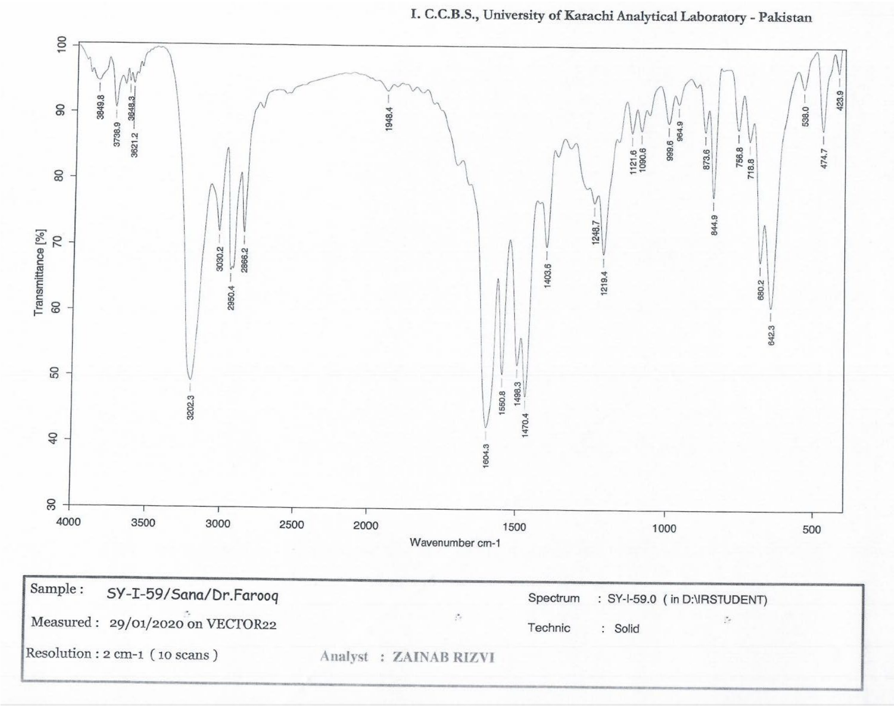

Figure S31. IR spectrum of *N*'-Hexanoylpyridine-4-carbohydrazide (**10**)

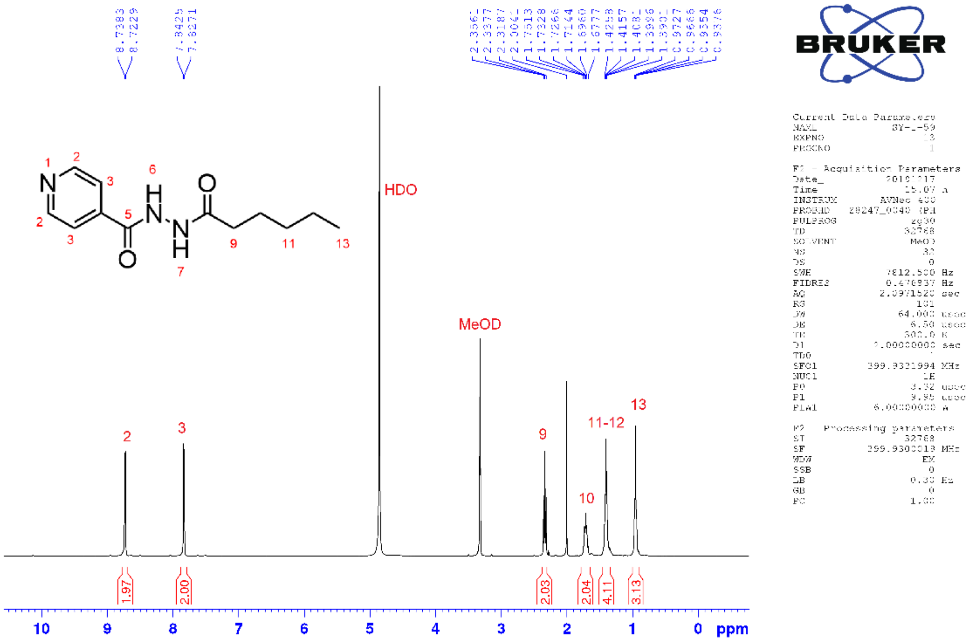

Figure S32. ^{1}H NMR spectrum of *N*'-Hexanoylpyridine-4-carbohydrazide (**10**)

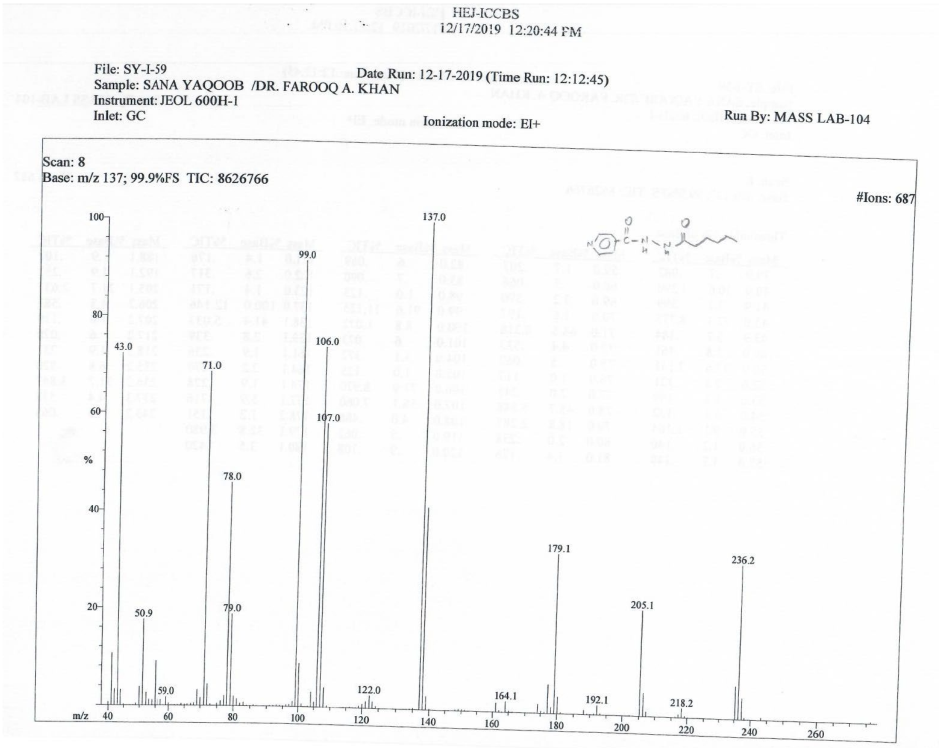

Figure S33. LR-MS (EI) spectrum of *N*'-Hexanoylpyridine-4-carbohydrazide (**10**)

File : D:\Data\sy-i-59-020120-c4.RAW
Full ms [49.500 - 700.500] - Range: 77.000 - 700.500
Scan No. 1 of 1

Mass	Relative Intensity	Theoretical Mass	Delta [ppm]	Delta [mmu]	RDB	Composition
77.9991	46.9	77.9980	14.3	1.1	5.5	$C_4 O_1 N_1$
80.0093	1.5	80.0110	-20.4	-1.6	0.0	$C_1 H_4 O_4$
81.0305	1.5	81.0327	-26.9	-2.2	4.0	$C_3 H_3 N_3$
82.0288	3.3	82.0293	-6.1	-0.5	3.5	$C_4 H_4 O_1 N_1$
91.0400	1.9	91.0395	5.6	0.5	0.5	$C_3 H_7 O_3$
		91.0382	20.4	1.9	1.0	$C_1 H_5 O_2 N_3$
		91.0422	-23.8	-2.2	5.0	$C_6 H_5 N_1$
96.0809	3.2	96.0813	-4.2	-0.4	2.5	$C_6 H_{10} N_1$
99.0795	75.6	99.0796	-1.1	-0.1	2.0	$C_4 H_9 N_3$
		99.0810	-14.7	-1.5	1.5	$C_6 H_{11} O_1$
100.0823	4.7					
103.0285	1.6	103.0296	-11.0	-1.1	6.5	$C_6 H_3 N_2$
		103.0269	15.0	1.5	2.0	$C_3 H_5 O_3 N_1$
104.0370	30.7	104.0374	-4.6	-0.5	6.0	$C_6 H_4 N_2$
		104.0348	21.1	2.2	1.5	$C_3 H_6 O_3 N_1$
105.0413	3.7	105.0426	-12.3	-1.3	1.0	$C_3 H_7 O_3 N_1$
106.0292	66.8	106.0293	-0.9	-0.1	5.5	$C_6 H_4 O_1 N_1$
		106.0266	24.4	2.6	1.0	$C_3 H_6 O_4$
107.0361	33.7	107.0371	-9.1	-1.0	5.0	$C_6 H_5 O_1 N_1$
		107.0344	16.0	1.7	0.5	$C_3 H_7 O_4$
		107.0331	28.5	3.1	1.0	$C_1 H_5 O_3 N_3$
108.0401	2.4	108.0409	-7.7	-0.8	0.5	$C_1 H_6 O_3 N_3$
		108.0423	-20.2	-2.2	0.0	$C_3 H_8 O_4$
119.0479	6.9	119.0483	-3.5	-0.4	6.0	$C_6 H_5 N_3$
		119.0497	-14.8	-1.8	5.5	$C_8 H_7 O_1$
		119.0457	19.0	2.3	1.5	$C_3 H_7 O_3 N_2$
120.0535	2.9	120.0535	-0.3	-0.0	1.0	$C_3 H_8 O_3 N_2$
		120.0562	-22.7	-2.7	5.5	$C_6 H_6 N_3$
121.0413	1.1	121.0402	9.0	1.1	5.5	$C_6 H_5 O_1 N_2$
122.0471	2.9	122.0480	-7.6	-0.9	5.0	$C_6 H_6 O_1 N_2$
		122.0453	14.4	1.8	0.5	$C_3 H_8 O_4 N_1$
123.0326	5.2	123.0320	4.3	0.5	5.0	$C_6 H_5 O_2 N_1$
		123.0293	26.1	3.2	0.5	$C_3 H_7 O_5$
137.0595	100.0	137.0589	4.2	0.6	5.0	$C_6 H_7 O_1 N_3$
		137.0603	-5.6	-0.8	4.5	$C_8 H_9 O_2$
		137.0562	23.8	3.3	0.5	$C_3 H_9 O_4 N_2$
138.0644	14.9	138.0641	2.2	0.3	0.0	$C_3 H_{10} O_4 N_2$
		138.0667	-17.2	-2.4	4.5	$C_6 H_8 O_1 N_3$
		138.0681	-26.9	-3.7	4.0	$C_8 H_{10} O_2$
139.0667	1.2	139.0633	23.9	3.3	4.0	$C_7 H_9 O_2 N_1$
161.0583	38.1	161.0589	-4.0	-0.6	7.0	$C_8 H_7 O_1 N_3$
		161.0603	-12.3	-2.0	6.5	$C_{10} H_9 O_2$
		161.0562	12.6	2.0	2.5	$C_5 H_9 O_4 N_2$
162.0615	3.7	162.0641	-16.0	-2.6	2.0	$C_5 H_{10} O_4 N_2$
174.0660	31.9	174.0667	-4.3	-0.7	7.5	$C_9 H_8 O_1 N_3$
		174.0641	11.1	1.9	3.0	$C_6 H_{10} O_4 N_2$
		174.0681	-12.0	-2.1	7.0	$C_{11} H_{10} O_2$
175.0737	6.8	175.0746	-4.9	-0.8	7.0	$C_9 H_9 O_1 N_3$
		175.0719	10.5	1.8	2.5	$C_6 H_{11} O_4 N_2$
		175.0759	-12.5	-2.2	6.5	$C_{11} H_{11} O_2$
177.1152	2.7	177.1154	-1.0	-0.2	5.0	$C_{11} H_{15} O_1 N_1$
		177.1127	14.1	2.5	0.5	$C_8 H_{17} O_4$
		177.1113	21.7	3.8	1.0	$C_6 H_{15} O_3 N_3$
179.0699	11.8	179.0695	2.3	0.4	6.0	$C_8 H_9 O_2 N_3$
		179.0708	-5.2	-0.9	5.5	$C_{10} H_{11} O_3$
		179.0668	17.3	3.1	1.5	$C_5 H_{11} O_5 N_2$
		179.0735	-20.1	-3.6	10.0	$C_{13} H_9 N_1$
180.0717	1.2	180.0687	16.2	2.9	10.0	$C_{12} H_8 N_2$
		180.0746	-16.4	-3.0	1.0	$C_5 H_{12} O_5 N_2$
188.0814	9.5	188.0824	-5.4	-1.0	7.5	$C_{10} H_{10} O_1 N_3$
		188.0797	8.8	1.7	3.0	$C_7 H_{12} O_4 N_2$
		188.0837	-12.6	-2.4	7.0	$C_{12} H_{12} O_2$
189.0871	1.7	189.0875	-2.2	-0.4	2.5	$C_7 H_{13} O_4 N_2$
		189.0902	-16.3	-3.1	7.0	$C_{10} H_{11} O_1 N_3$
		189.0916	-23.4	-4.4	6.5	$C_{12} H_{13} O_2$
205.1090	7.2	205.1103	-6.5	-1.3	6.0	$C_{12} H_{15} O_2 N_1$
		205.1076	6.6	1.4	1.5	$C_9 H_{17} O_5$

Mass	Relative Intensity	Theoretical Mass	Delta [ppm]	Delta [mmu]	RDB	Composition
		205.1063	13.2	2.7	2.0	$C_7 H_{15} O_4 N_3$
206.1075	1.6	206.1055	9.6	2.0	6.0	$C_{11} H_{14} O_2 N_2$
		206.1096	-9.9	-2.0	10.0	$C_{16} H_{14}$
		206.1028	22.6	4.7	1.5	$C_8 H_{16} O_5 N_1$
217.1225	3.1	217.1229	-1.6	-0.4	6.5	$C_{14} H_{17} O_2$
		217.1215	4.5	1.0	7.0	$C_{12} H_{15} O_1 N_3$
		217.1188	16.9	3.7	2.5	$C_9 H_{17} O_4 N_2$
235.1312	1.8	235.1321	-3.8	-0.9	6.0	$C_{12} H_{17} O_2 N_3$
		235.1294	7.6	1.8	1.5	$C_9 H_{19} O_5 N_2$
		235.1334	-9.5	-2.2	5.5	$C_{14} H_{19} O_3$
		235.1361	-20.9	-4.9	10.0	$C_{17} H_{17} N_1$

Figure S34. HR-MS (EI) spectral data of *N'*-Hexanoylpyridine-4-carbohydrazide (**10**)

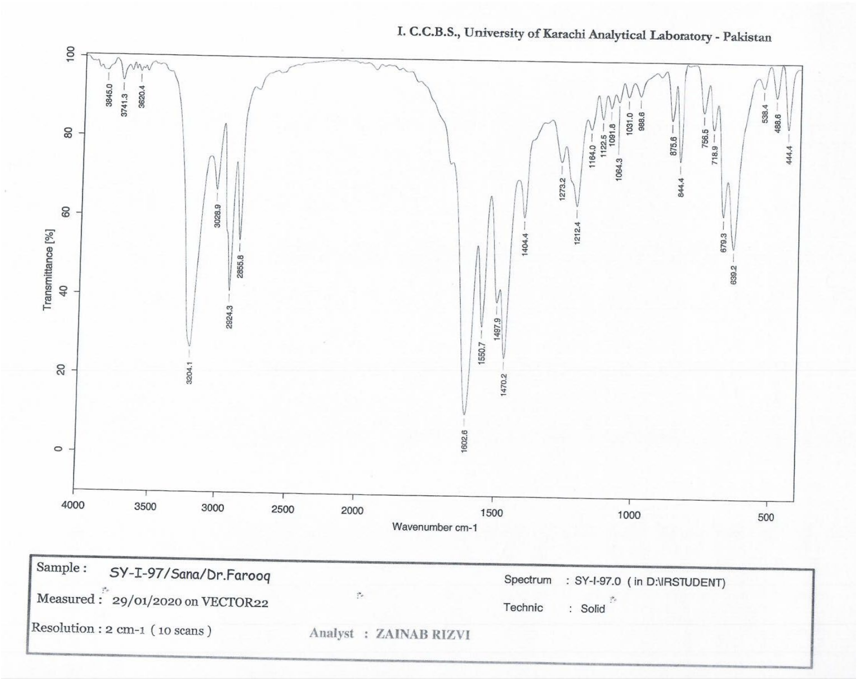

Figure S35. IR spectrum of *N'*-Octanoylpyridine-4-carbohydrazide (**11**)

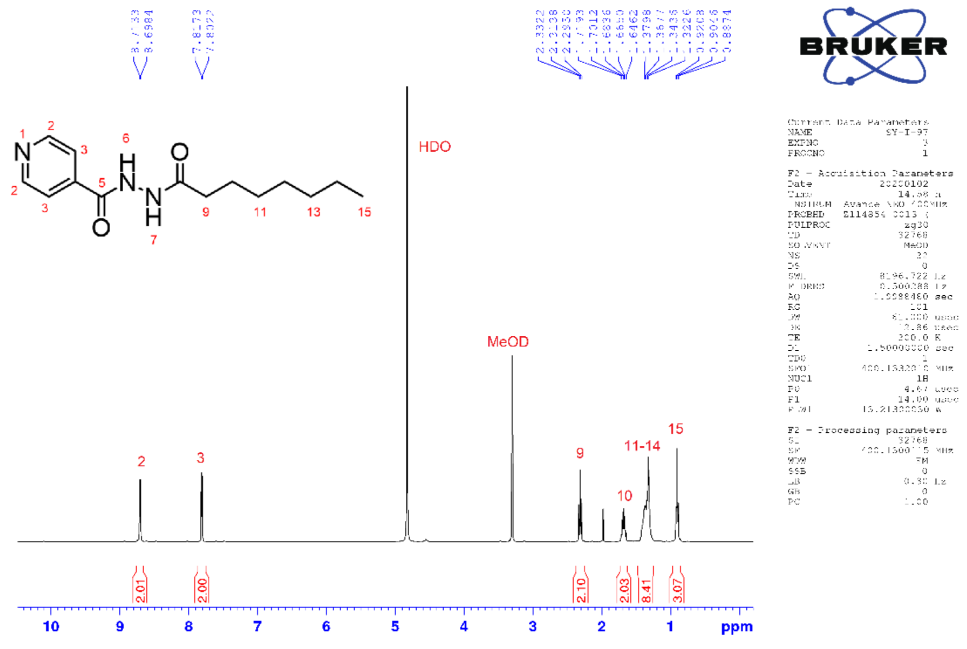

Figure S36. ^{1}H NMR spectrum of *N'*-Octanoylpyridine-4-carbohydrazide (**11**)

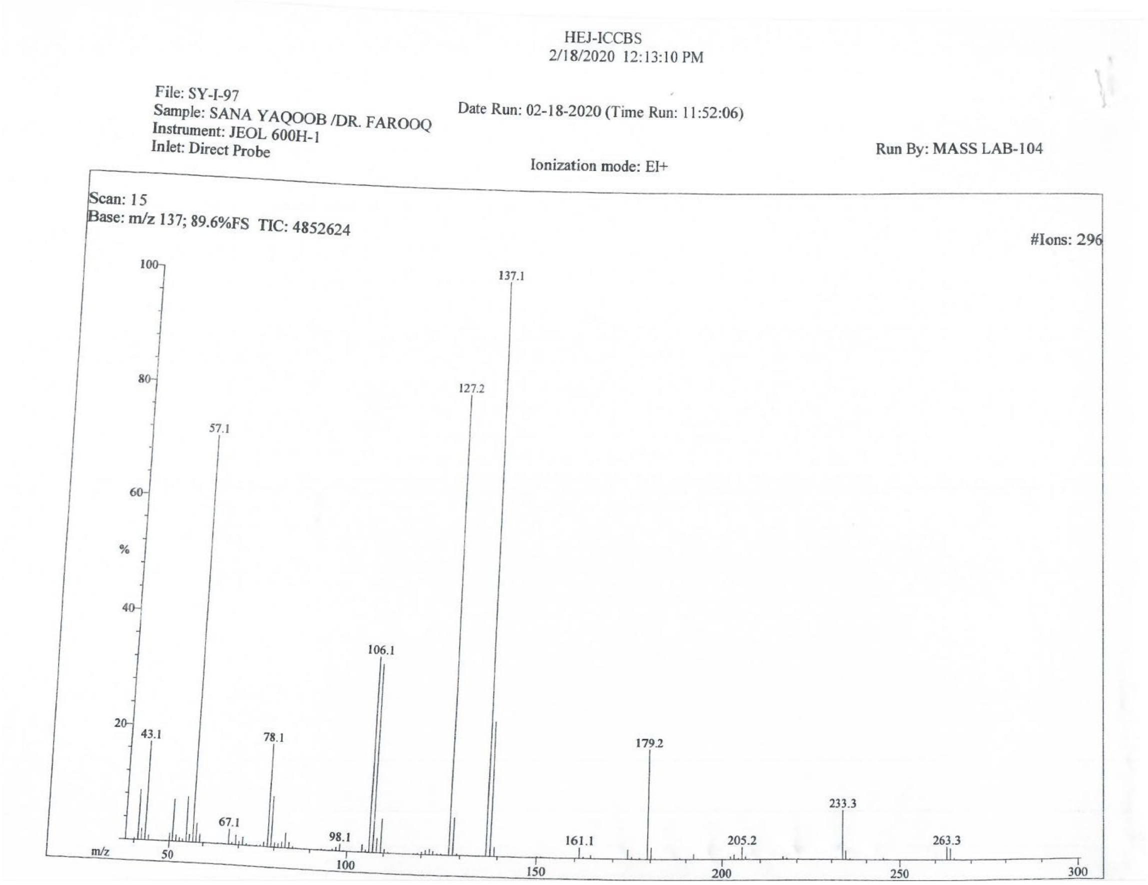

Figure S37. LR-MS (EI) spectrum of *N*'-Octanoylpyridine-4-carbohydrazide (**11**)

File : D:\Data\sy-i-97-130320-c9.RAW
Full ms [49.500 - 700.500] - Range: 92.000 - 265.000
Scan No. 1 of 1

Mass	Relative Intensity	Theoretical Mass	Delta [ppm]	Delta [mmu]	RDB	Composition
92.0357	0.6	92.0348	9.9	0.9	0.5	$C_2 H_6 O_3 N_1$
		92.0374	-19.2	-1.8	5.0	$C_5 H_4 N_2$
93.0694	0.8	93.0704	-10.8	-1.0	3.5	$C_7 H_9$
94.0703	0.7					
95.0867	1.8	95.0861	6.3	0.6	2.5	$C_7 H_{11}$
96.0859	2.1					
97.0678	1.2	97.0653	25.5	2.5	2.5	$C_6 H_9 O_1$
97.1001	2.7	97.1017	-16.7	-1.6	1.5	$C_7 H_{13}$
98.0724	2.6	98.0718	6.4	0.6	2.5	$C_4 H_8 N_3$
		98.0732	-7.3	-0.7	2.0	$C_6 H_{10} O_1$
98.1077	0.6	98.1096	-18.5	-1.8	1.0	$C_7 H_{14}$
99.0734	0.5					
100.0749	0.6	100.0762	-13.2	-1.3	1.5	$C_5 H_{10} O_1 N_1$
104.0383	5.3	104.0374	8.4	0.9	6.0	$C_6 H_4 N_2$
105.0461	1.7	105.0453	8.3	0.9	5.5	$C_6 H_5 N_2$
106.0290	42.0	106.0293	-2.9	-0.3	5.5	$C_6 H_4 O_1 N_1$
		106.0266	22.4	2.4	1.0	$C_3 H_6 O_4$
107.0366	31.2	107.0371	-4.3	-0.5	5.0	$C_6 H_5 O_1 N_1$
		107.0344	20.7	2.2	0.5	$C_3 H_7 O_4$
107.0849	0.6	107.0861	-11.2	-1.2	3.5	$C_8 H_{11}$
108.0414	2.5	108.0409	4.4	0.5	0.5	$C_1 H_6 O_3 N_3$
		108.0423	-8.1	-0.9	0.0	$C_3 H_8 O_4$
108.0870	0.5					
109.1017	8.3	109.1017	-0.7	-0.1	2.5	$C_8 H_{13}$
110.1037	1.7					
111.0816	0.6	111.0810	5.1	0.6	2.5	$C_7 H_{11} O_1$
		111.0796	17.2	1.9	3.0	$C_5 H_9 N_3$
111.1159	0.9	111.1174	-13.4	-1.5	1.5	$C_8 H_{15}$
112.0783	0.8	112.0762	18.5	2.1	2.5	$C_6 H_{10} O_1 N_1$
114.0921	0.8	114.0919	1.8	0.2	1.5	$C_6 H_{12} O_1 N_1$
118.0602	0.6	118.0617	-12.2	-1.4	1.5	$C_3 H_8 O_2 N_3$
		118.0630	-23.6	-2.8	1.0	$C_5 H_{10} O_3$
119.0480	4.4	119.0483	-3.1	-0.4	6.0	$C_6 H_5 N_3$
		119.0497	-14.4	-1.7	5.5	$C_8 H_7 O_1$
		119.0457	19.4	2.3	1.5	$C_3 H_7 O_3 N_2$
120.0544	3.3	120.0535	7.9	1.0	1.0	$C_3 H_8 O_3 N_2$
		120.0562	-14.4	-1.7	5.5	$C_6 H_6 N_3$
		120.0575	-25.6	-3.1	5.0	$C_8 H_8 O_1$
121.0417	1.3	121.0402	12.6	1.5	5.5	$C_6 H_5 O_1 N_2$
122.0482	3.0	122.0480	1.6	0.2	5.0	$C_6 H_6 O_1 N_2$
		122.0453	23.5	2.9	0.5	$C_3 H_8 O_4 N_1$
122.0981	1.0	122.0970	9.4	1.1	3.5	$C_8 H_{12} N_1$
123.0530	1.9	123.0532	-1.3	-0.2	0.0	$C_3 H_9 O_4 N_1$
		123.0558	-23.1	-2.8	4.5	$C_6 H_7 O_1 N_2$
123.1129	0.8					
124.1162	0.7	124.1126	29.0	3.6	2.5	$C_8 H_{14} N_1$
125.0968	0.6	125.0966	1.1	0.1	2.5	$C_8 H_{13} O_1$
		125.0953	11.8	1.5	3.0	$C_6 H_{11} N_3$
126.0941	1.1	126.0919	17.9	2.3	2.5	$C_7 H_{12} O_1 N_1$
127.1120	65.7	127.1123	-2.4	-0.3	1.5	$C_8 H_{15} O_1$
		127.1109	8.2	1.0	2.0	$C_6 H_{13} N_3$
128.1157	6.3	128.1188	-24.0	-3.1	1.5	$C_6 H_{14} N_3$
136.1133	0.7	136.1126	5.2	0.7	3.5	$C_9 H_{14} N_1$
137.0575	95.1	137.0562	9.3	1.3	0.5	$C_3 H_9 O_4 N_2$
		137.0589	-10.3	-1.4	5.0	$C_6 H_7 O_1 N_3$
		137.0603	-20.1	-2.7	4.5	$C_8 H_9 O_2$
138.0654	20.5	138.0641	9.5	1.3	0.0	$C_3 H_{10} O_4 N_2$
		138.0667	-9.9	-1.4	4.5	$C_6 H_8 O_1 N_3$
		138.0681	-19.6	-2.7	4.0	$C_8 H_{10} O_2$
139.0686	2.2					
148.0512	0.7	148.0511	0.9	0.1	6.5	$C_7 H_6 O_1 N_3$
		148.0524	-8.2	-1.2	6.0	$C_9 H_8 O_2$
		148.0484	19.0	2.8	2.0	$C_4 H_8 O_4 N_2$
149.0243	0.8	149.0239	3.0	0.4	6.5	$C_8 H_5 O_3$
		149.0225	12.0	1.8	7.0	$C_6 H_3 O_2 N_3$
		149.0265	-15.0	-2.2	11.0	$C_{11} H_3 N_1$
161.0593	28.4	161.0589	2.1	0.3	7.0	$C_8 H_7 O_1 N_3$

Mass	Relative Intensity	Theoretical Mass	Delta [ppm]	Delta [mmu]	RDB	Composition
		263.1607	8.0	2.1	1.5	$C_{11} H_{23} O_5 N_2$
		263.1674	-17.4	-4.6	10.0	$C_{19} H_{21} N_1$
264.1705	0.8	264.1712	-2.8	-0.7	5.5	$C_{14} H_{22} O_2 N_3$
		264.1685	7.3	1.9	1.0	$C_{11} H_{24} O_5 N_2$
		264.1725	-7.9	-2.1	5.0	$C_{16} H_{24} O_3$
		264.1752	-18.1	-4.8	9.5	$C_{19} H_{22} N_1$
		264.1626	29.5	7.8	10.0	$C_{18} H_{20} N_2$

Figure S38. HR-MS (EI) spectral data of *N*'-Octanoylpyridine-4-carbohydrazide (**11**)

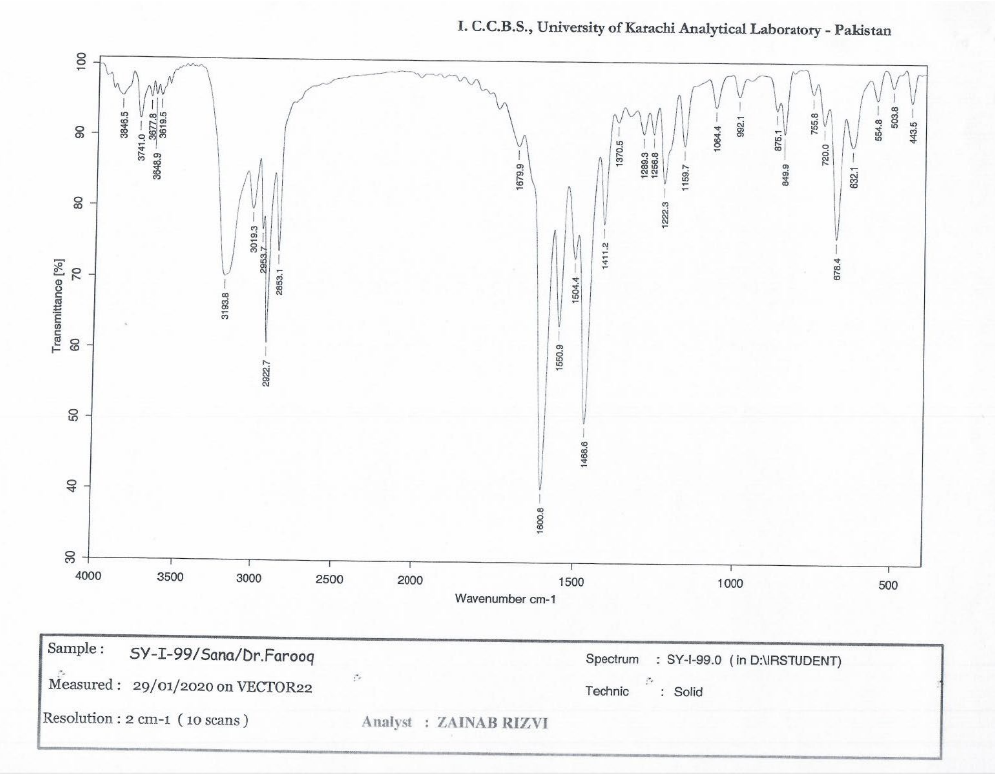

Figure S39. IR spectrum of *N*'-Decanoylpyridine-4-carbohydrazide (**12**)

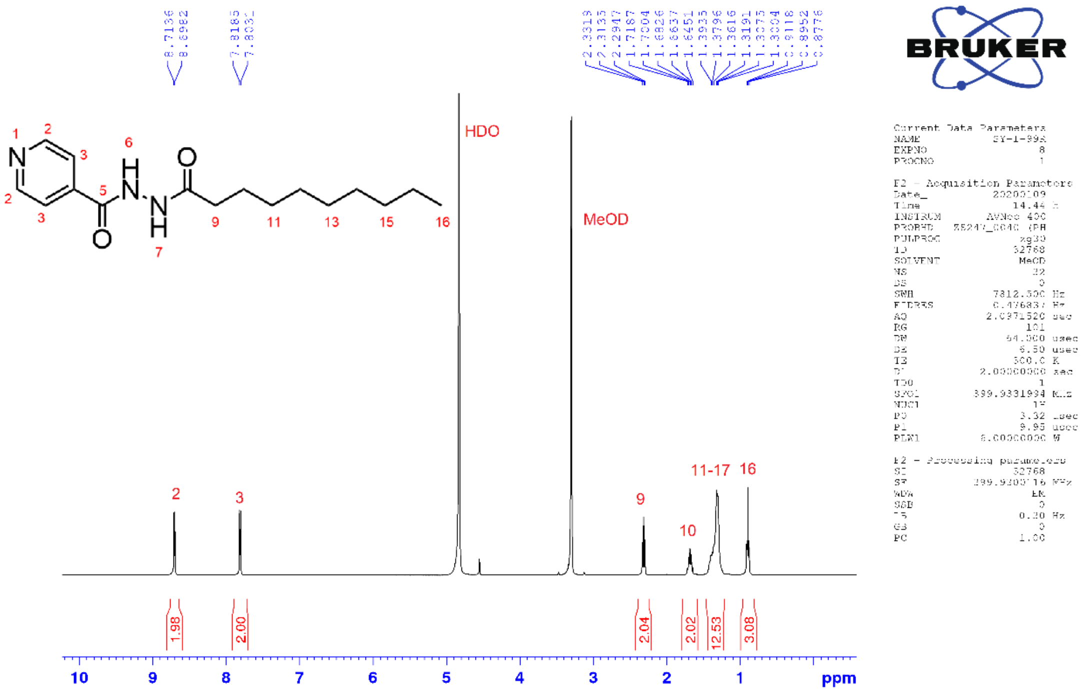

Figure S40. ^{1}H NMR spectrum of *N*'-Decanoylpyridine-4-carbohydrazide (**12**)

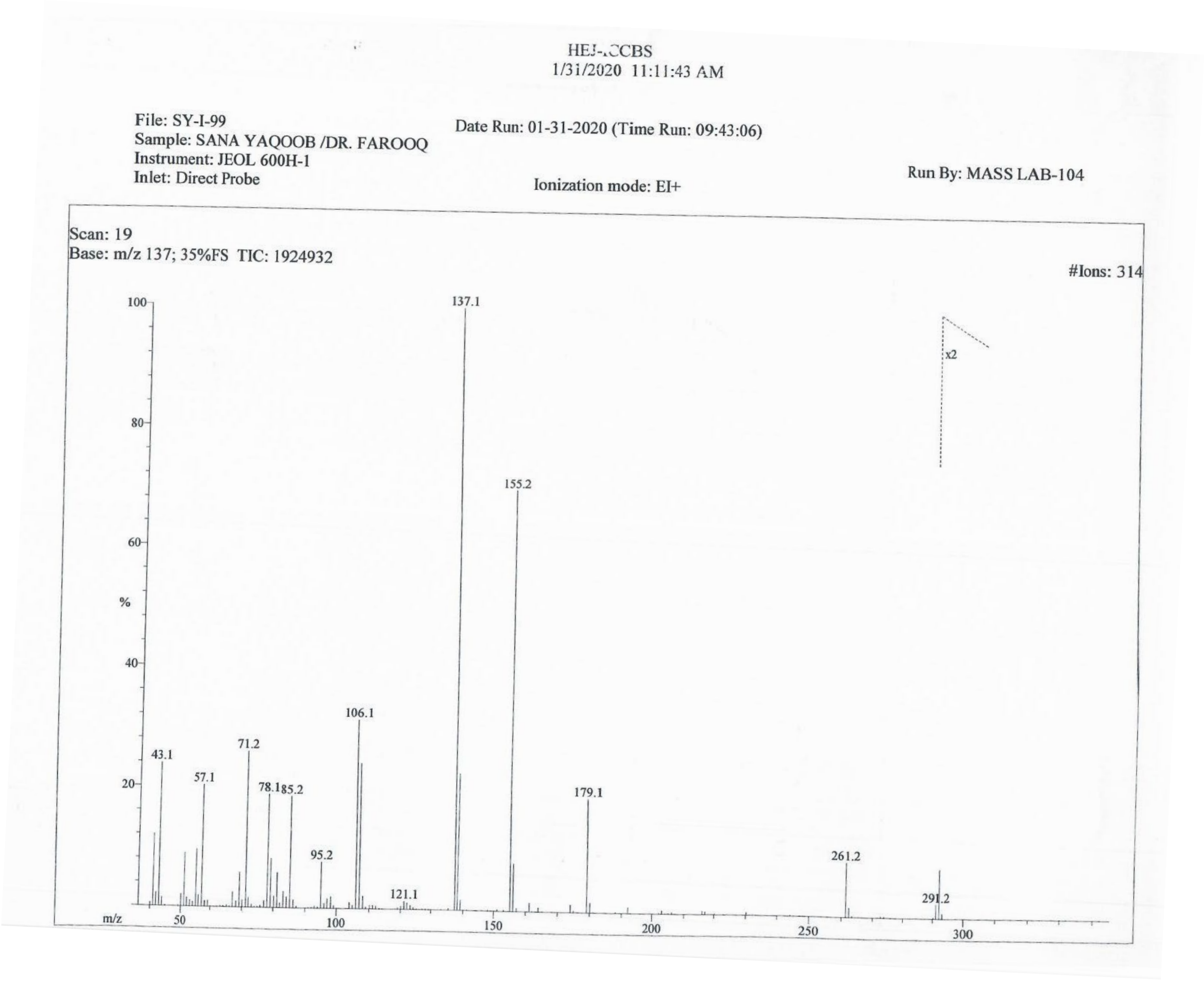

Figure S41. LR-MS (EI) spectrum of *N*'-Decanoylpyridine-4-carbohydrazide (**12**)

File : D:\Data\sy-i-99_180220-c13.RAW
Full ms [49.500 - 750.500] - Range: 101.000 - 293.000
Scan No. 1 of 1

Mass	Relative Intensity	Theoretical Mass	Delta [ppm]	Delta [mmu]	RDB	Composition
101.0157	0.1					
101.0573	0.2	101.0603	-28.9	-2.9	1.5	$C_5 H_9 O_2$
102.0296	0.2	102.0304	-7.4	-0.8	2.5	$C_2 H_4 O_2 N_3$
		102.0317	-20.6	-2.1	2.0	$C_4 H_6 O_3$
102.0447	0.2	102.0429	17.5	1.8	2.0	$C_3 H_6 O_2 N_2$
102.0678	0.0	102.0681	-3.0	-0.3	1.0	$C_5 H_{10} O_2$
103.0296	1.8	103.0269	25.6	2.6	2.0	$C_3 H_5 O_3 N_1$
104.0368	28.1	104.0348	19.5	2.0	1.5	$C_3 H_6 O_3 N_1$
104.0990	0.0					
105.0435	5.2	105.0426	8.7	0.9	1.0	$C_3 H_7 O_3 N_1$
		105.0413	21.5	2.3	1.5	$C_1 H_5 O_2 N_4$
106.0288	23.7	106.0266	20.9	2.2	1.0	$C_3 H_6 O_4$
106.5438	0.0					
107.0351	6.7	107.0344	6.4	0.7	0.5	$C_3 H_7 O_4$
		107.0331	18.9	2.0	1.0	$C_1 H_5 O_3 N_3$
107.0835	0.8					
107.5522	0.1					
108.0407	0.8	108.0409	-2.1	-0.2	0.5	$C_1 H_6 O_3 N_3$
		108.0423	-14.5	-1.6	0.0	$C_3 H_8 O_4$
108.0811	1.2					
109.0541	0.4					
109.0926	0.7					
110.0077	0.0					
110.0116	0.0	110.0116	-0.3	-0.0	5.0	$C_4 H_2 O_2 N_2$
110.0565	0.3					
110.0969	7.0					
111.1025	1.9					
112.0330	0.0					
112.0820	0.6					
112.1229	0.4					
113.0380	0.1	113.0351	25.7	2.9	3.5	$C_4 H_5 O_2 N_2$
113.0866	0.2					
113.1302	0.1					
114.0437	0.1	114.0429	6.5	0.7	3.0	$C_4 H_6 O_2 N_2$
114.0930	0.2					
114.1329	0.0					
114.5609	0.0					
115.0583	0.5					
115.0747	0.5	115.0759	-10.4	-1.2	1.5	$C_6 H_{11} O_2$
116.0544	0.5					
117.0607	1.0					
118.0620	1.7	118.0617	2.6	0.3	1.5	$C_3 H_8 O_2 N_3$
		118.0630	-8.7	-1.0	1.0	$C_5 H_{10} O_3$
119.0484	11.3	119.0457	22.7	2.7	1.5	$C_3 H_7 O_3 N_2$
120.0516	10.1	120.0535	-16.1	-1.9	1.0	$C_3 H_8 O_3 N_2$
121.0444	1.3					
121.0877	0.4	121.0851	21.2	2.6	0.0	$C_3 H_{11} O_2 N_3$
122.0341	6.4	122.0328	11.1	1.4	1.0	$C_2 H_6 O_4 N_2$
		122.0368	-21.8	-2.7	5.0	$C_7 H_6 O_2$
122.0825	1.8	122.0804	17.4	2.1	0.0	$C_2 H_{10} O_2 N_4$
123.0139	4.8	123.0154	-12.2	-1.5	1.5	$H_3 O_4 N_4$
123.0814	0.5					
123.9692	0.1					
124.0109	0.4					
124.0335	0.4	124.0358	-19.1	-2.4	0.5	$C_1 H_6 O_4 N_3$
124.0862	3.4					
124.9688	0.0					
125.0160	0.1					
125.0847	0.5					
126.0065	0.0	126.0065	-0.2	-0.0	5.0	$C_4 H_2 O_3 N_2$
126.0531	0.2	126.0555	-19.4	-2.4	3.5	$C_6 H_8 O_2 N_1$
126.0993	0.1					
126.8628	0.0					
127.0018	0.2	127.0018	0.4	0.1	5.0	$C_3 H_1 O_3 N_3$
		127.0031	-10.1	-1.3	4.5	$C_5 H_3 O_4$
127.0631	0.1	127.0633	-1.7	-0.2	3.0	$C_6 H_9 O_2 N_1$
127.1032	0.0					

Mass	Relative Intensity	Theoretical Mass	Delta [ppm]	Delta [mmu]	RDB	Composition
272.1744	1.6	272.1736	2.9	0.8	3.0	$C_{13} H_{24} O_4 N_2$
		272.1776	-11.8	-3.2	7.0	$C_{18} H_{24} O_2$
273.0768	0.0	273.0750	6.9	1.9	10.0	$C_{13} H_{11} O_4 N_3$
		273.0790	-7.8	-2.1	14.0	$C_{18} H_{11} O_2 N_1$
273.1810	4.6	273.1814	-1.7	-0.5	2.5	$C_{13} H_{25} O_4 N_2$
		273.1855	-16.4	-4.5	6.5	$C_{18} H_{25} O_2$
		273.1729	29.6	8.1	7.0	$C_{17} H_{23} O_2 N_1$
274.1871	1.0	274.1893	-7.9	-2.2	2.0	$C_{13} H_{26} O_4 N_2$
		274.1933	-22.6	-6.2	6.0	$C_{18} H_{26} O_2$
		274.1807	23.3	6.4	6.5	$C_{17} H_{24} O_2 N_1$
275.0749	0.0	275.0780	-11.3	-3.1	9.5	$C_{12} H_{11} O_4 N_4$
		275.0708	14.9	4.1	13.5	$C_{18} H_{11} O_3$
		275.0695	19.8	5.4	14.0	$C_{16} H_9 O_2 N_3$
		275.0821	-25.9	-7.1	13.5	$C_{17} H_{11} O_2 N_2$
275.0920	0.0	275.0906	4.9	1.4	9.0	$C_{13} H_{13} O_4 N_3$
		275.0946	-9.7	-2.7	13.0	$C_{18} H_{13} O_2 N_1$
275.1092	0.0	275.1072	7.1	2.0	12.5	$C_{19} H_{15} O_2$
		275.1144	-19.1	-5.3	8.5	$C_{13} H_{15} O_3 N_4$
		275.1032	21.7	6.0	8.5	$C_{14} H_{15} O_4 N_2$
		275.1158	-24.0	-6.6	8.0	$C_{15} H_{17} O_4 N_1$
275.1813	0.1	275.1845	-11.8	-3.2	2.0	$C_{12} H_{25} O_4 N_3$
		275.1760	19.3	5.3	6.5	$C_{16} H_{23} O_2 N_2$
		275.1885	-26.4	-7.3	6.0	$C_{17} H_{25} O_2 N_1$
276.1808	0.0	276.1798	3.9	1.1	2.0	$C_{11} H_{24} O_4 N_4$
		276.1838	-10.7	-2.9	6.0	$C_{16} H_{24} O_2 N_2$
		276.1725	30.0	8.3	6.0	$C_{17} H_{24} O_3$
277.0921	0.0	277.0937	-5.7	-1.6	8.5	$C_{12} H_{13} O_4 N_4$
		277.0977	-20.2	-5.6	12.5	$C_{17} H_{13} O_2 N_2$
		277.0865	20.4	5.6	12.5	$C_{18} H_{13} O_3$
		277.0851	25.2	7.0	13.0	$C_{16} H_{11} O_2 N_3$
280.0901	0.0	280.0848	18.9	5.3	12.0	$C_{16} H_{12} O_3 N_2$
		280.0960	-21.2	-6.0	12.0	$C_{15} H_{12} O_2 N_4$
		280.0974	-26.0	-7.3	11.5	$C_{17} H_{14} O_3 N_1$
280.1076	0.0	280.1086	-3.5	-1.0	11.5	$C_{16} H_{14} O_2 N_3$
		280.1099	-8.3	-2.3	11.0	$C_{18} H_{16} O_3$
281.1102	0.0	281.1052	17.7	5.0	11.0	$C_{17} H_{15} O_3 N_1$
		281.1164	-22.2	-6.3	11.0	$C_{16} H_{15} O_2 N_3$
		281.1039	22.5	6.3	11.5	$C_{15} H_{13} O_2 N_4$
		281.1178	-27.0	-7.6	10.5	$C_{18} H_{17} O_3$
282.0932	0.0	282.0892	14.1	4.0	11.0	$C_{17} H_{14} O_4$
		282.0879	18.9	5.3	11.5	$C_{15} H_{12} O_3 N_3$
		282.1004	-25.7	-7.2	11.0	$C_{16} H_{14} O_3 N_2$
282.1511	0.0	282.1494	5.9	1.7	9.5	$C_{18} H_{20} O_2 N_1$
		282.1454	20.1	5.7	5.5	$C_{13} H_{20} O_4 N_3$
		282.1580	-24.4	-6.9	5.0	$C_{14} H_{22} O_4 N_2$
283.1167	0.0	283.1195	-9.9	-2.8	10.5	$C_{15} H_{15} O_2 N_4$
		283.1208	-14.6	-4.1	10.0	$C_{17} H_{17} O_3 N_1$
		283.1083	29.8	8.4	10.5	$C_{16} H_{15} O_3 N_2$
285.1782	0.0	285.1814	-11.4	-3.3	3.5	$C_{14} H_{25} O_4 N_2$
		285.1729	18.6	5.3	8.0	$C_{18} H_{23} O_2 N_1$
		285.1855	-25.5	-7.3	7.5	$C_{19} H_{25} O_2$
285.2029	0.0	285.2052	-8.1	-2.3	3.0	$C_{14} H_{27} O_3 N_3$
		285.2066	-12.8	-3.6	2.5	$C_{16} H_{29} O_4$
287.1943	0.0	287.1971	-9.6	-2.7	2.5	$C_{14} H_{27} O_4 N_2$
		287.1885	20.2	5.8	7.0	$C_{18} H_{25} O_2 N_1$
		287.2011	-23.6	-6.8	6.5	$C_{19} H_{27} O_2$
288.2010	0.0	288.2049	-13.5	-3.9	2.0	$C_{14} H_{28} O_4 N_2$
		288.1964	16.2	4.7	6.5	$C_{18} H_{26} O_2 N_1$
		288.2089	-27.5	-7.9	6.0	$C_{19} H_{28} O_2$
289.1835	0.0	289.1804	10.9	3.2	6.5	$C_{18} H_{25} O_3$
		289.1876	-14.0	-4.0	2.5	$C_{12} H_{25} O_4 N_4$
		289.1790	15.6	4.5	7.0	$C_{16} H_{23} O_2 N_3$
		289.1916	-27.9	-8.1	6.5	$C_{17} H_{25} O_2 N_2$
290.0699	0.0	290.0691	2.8	0.8	14.0	$C_{17} H_{10} O_3 N_2$
290.1898	0.0	290.1882	5.5	1.6	6.0	$C_{18} H_{26} O_3$
		290.1869	10.1	2.9	6.5	$C_{16} H_{24} O_2 N_3$
		290.1954	-19.4	-5.6	2.0	$C_{12} H_{26} O_4 N_4$
291.1937	0.1	291.1947	-3.2	-0.9	6.0	$C_{16} H_{25} O_2 N_3$
		291.1960	-7.8	-2.3	5.5	$C_{18} H_{27} O_3$
292.2009	0.0	292.2025	-5.5	-1.6	5.5	$C_{16} H_{26} O_2 N_3$

Figure S42. HR-MS (EI) spectral data of *N'*-Decanoylpyridine-4-carbohydrazide (**12**)

Table S1. Time-kill assay of compound **4** against the *Pseudomonas aeruginosa* PA-01 observed onto nutrient agar plates *via* counting log CFU/mL (± S.D), after 24 hours

Time (h)	MIC 0.5X (4 µg)	MIC 1X (8 µg)	MIC 2X (16 µg)	Ampicillin/Cloxacillin MIC 1X (32 µg)	*Pseudomonas aeruginosa* PA-01
0	6± 0.19	6± 0.16	6± 0.19	6± 0.26	6± 0.21
4	6± 0.13	5± 0.19	2± 0.22	5± 0.29	6± 0.19
8	5.2± 0.18	3.2± 0.18	1± 0.27	5± 0.31	6.8± 0.21
12	3± 0.179	2± 0.21	1± 0.27	3± 0.37	7.3± 0.16
24	2± 0.18	1.1± 0.19	1± 0.24	2± 0.32	7.2± 0.18
48	1.1± ± 0.17	0.8± 0.16	1± 0.19	1± 0.36	7.4± 0.18

Table S2. Time-kill assay of compound **4** against *the Staphylococcus aureus* ATCC 29213 observed onto nutrient agar plates *via* counting log CFU/mL (± S.D), after 24 hours

Time (h)	MIC 0.5X (4 µg)	MIC 1X (8 µg)	MIC 2X (16 µg)	ampicillin/cloxacillin MIC 1X (16 µg)	*Staphylococcus aureus ATCC* 29213
0	6± 0.61	6± 0.58	6± 0.47	6± 0.54	6± 0.69
4	5± 0.63	5.8± 0.60	4.91± 0.45	5.8± 0.51	5.9± 0.81
8	4.8± 0.58	4.3± 0.57	3.1± 0.41	5.3± 0.49	6.3± 0.84
12	4.67± 0.54	3.51± 0.51	2.24± 0.44	3.1± 0.53	6.8± 0.79
24	3.9± 0.56	2.9± 0.43	1.2± 0.4	2.9± 0.55	7.1± 0.89
48	4.12± 0.59	1.78± 0.51	1± 0.52	1.9± 0.56	6.9± 1.17

Table S3. Time-kill assay of compound **6** against the *Candida glabrata* ATCC 2001 observed onto SDA agar plates *via* counting log CFU/mL (± S.D), after 48 hours

Time (h)	MIC 0.5X (8 µg)	MIC 1X (16 µg)	MIC 2X (32 µg)	Fluconazole (32 µg)	*Candida glabrata* ATCC 2001
0	5.88± 0.19	5.93 ± 0.16	5.98± 0.21	5.71± 0.19	5.75 ± 0.18
4	5.59± 0.21	4.98± 0.24	5.02± 0.19	5.7± 0.32	5.75 ± 0.21
8	5.31± 0.17	4.57± 0.21	4.11± 0.22	5.62± 0.67	6.1± 0.16
12	4.92± 0.11	4.02 ± 0.13	3.02± 0.17	4.01± 0.81	6.89 ± 0.12
24	3.96± 0.13	3.21± 0.18	2.11± 0.19	3.15± 1.01	7.02± 0.13
48	3.01± 0.09	2.01± 0.19	1.12± 1.08	2.81± 1.32	7.04± 0.16

Table S4. Comparison of percent hemolysis % (± S.D) observed for the compounds **4** and **6**

Time (h)	Ampicillin/cloxacillin (32 µg)	Fluconazole (32 µg)	Compound **4** (32 µg)	Compound **6** (32 µg)	DMSO (5%)	Triton X-100 (1%)	SDS (1%)
0	0	0	0	0	0	3± 0.11	5± 0.19
5	0	0	0	0	0	20± 0.11	25± 0.44
10	0	0	0	0	10± 0.39	60± 0.58	60± 0.61
15	0	0	0	0	15± 0.46	80± 0.57	80± 0.62
20	0	1± 0.12	0	2.6± 0.42	22± 0.46	90± 0.61	100± 0.64
30	0	1± 0.19	0	2.8± 0.44	27± 0.56	100± 0.65	100± 0.79
